# G protein‐biased kratom‐alkaloids and synthetic carfentanil‐amide opioids as potential treatments for alcohol use disorder

**DOI:** 10.1111/bph.14913

**Published:** 2020-01-24

**Authors:** Anna M. Gutridge, Meridith T. Robins, Robert J. Cassell, Rajendra Uprety, Kendall L. Mores, Mee Jung Ko, Gavril W. Pasternak, Susruta Majumdar, Richard M. van Rijn

**Affiliations:** ^1^ Department of Medicinal Chemistry and Molecular Pharmacology, College of Pharmacy Purdue University West Lafayette Indiana; ^2^ Purdue Institute for Drug Discovery Purdue University West Lafayette Indiana; ^3^ Purdue Institute for Integrative Neuroscience Purdue University West Lafayette Indiana; ^4^ Purdue Interdisciplinary Life Sciences Graduate Program Purdue University West Lafayette Indiana; ^5^ Department of Neurology and Molecular Pharmacology Memorial Sloan Kettering Cancer Center New York New York; ^6^ Center for Clinical Pharmacology St. Louis College of Pharmacy and Washington University School of Medicine St. Louis Missouri

## Abstract

**Background and Purpose:**

*Mitragyna speciosa*, more commonly known as kratom, is a plant that contains opioidergic alkaloids but is unregulated in most countries. Kratom is used in the self‐medication of chronic pain and to reduce illicit and prescription opioid dependence. Kratom may be less dangerous than typical opioids because of the stronger preference of kratom alkaloids to induce receptor interaction with G proteins over β‐arrestin proteins. We hypothesized that kratom (alkaloids) can also reduce alcohol intake.

**Experimental Approach:**

We pharmacologically characterized kratom extracts, kratom alkaloids (mitragynine, 7‐hydroxymitragynine, paynantheine, and speciogynine) and synthetic carfentanil‐amide opioids for their ability to interact with G proteins and β‐arrestin at μ, δ, and κ opioid receptors *in vitro.* We used C57BL/6 mice to assess to which degree these opioids could reduce alcohol intake and whether they had rewarding properties.

**Key Results:**

Kratom alkaloids were strongly G protein‐biased at all three opioid receptors and reduced alcohol intake, but kratom and 7‐hydroxymitragynine were rewarding. Several results indicated a key role for δ opioid receptors, including that the synthetic carfentanil‐amide opioid MP102—a G protein‐biased agonist with modest selectivity for δ opioid receptors—reduced alcohol intake, whereas the G protein‐biased μ opioid agonist TRV130 did not.

**Conclusion and Implications:**

Our results suggest that kratom extracts can decrease alcohol intake but still carry significant risk upon prolonged use. Development of more δ opioid‐selective synthetic opioids may provide a safer option than kratom to treat alcohol use disorder with fewer side effects.

## Graphical abstract

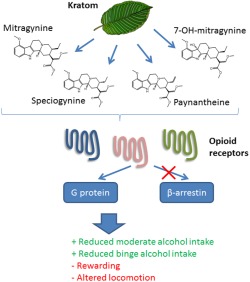

**Abbreviations**
μOP: μ opioid receptor
δOP: δ opioid receptor
κOP: κ opioid receptor
DAMGO: (2*S*)‐2‐[[2‐[[(2*R*)‐2‐[[(2*S*)‐2‐Amino‐3‐(4‐hydroxyphenyl)propanoyl]amino]propanoyl] amino]acetyl]‐methylamino]‐*N*‐(2‐hydroxyethyl)‐3‐phenylpropanamide
TRV130: (3‐Methoxythiophen‐2‐yl)methyl]((2‐[(9*R*)‐9‐(pyridin‐2‐yl)‐6‐oxaspiro‐[4.5]decan‐9‐yl]ethyl))amine
U50,488: 2‐(3,4‐dichlorophenyl)‐*N*‐methyl‐*N*‐[(1*R*,2*R*)‐2‐pyrrolidin‐1‐ylcyclohexyl]acetamide
MP102: *N*‐cycloheptyl‐1‐phenethyl‐4‐(*N*‐phenylpropionamido)piperidine‐4‐carboxamide
MP103: *N*‐cyclopropyl‐1‐phenethyl‐4‐(*N*‐phenylpropionamido)piperidine‐4‐carboxamide
MP105: *N*‐(tert‐butyl)‐1‐phenethyl‐4‐(*N*‐phenylpropionamido)piperidine‐4‐carboxamide
U‐2 OS: U‐2 osteosarcoma
MC: multiple comparison
CPP: conditioned place preference

**What is already known**

- Kratom is an opioid‐containing plant that is legal in most countries around the world.
- Kratom alkaloids display a preference for G protein signalling over β‐arrestin recruitment.

**What does this study add**

- Kratom extract, opioid alkaloids. and synthetic G protein‐biased opioids reduce alcohol intake in mice.
- The complex pharmacology of kratom alkaloids may explain adverse effects on general locomotion and reward.

**What is the clinical significance**

- Self‐medication of alcohol use disorder with kratom is possible but not without risks.
- δ Opioid receptor‐selective G protein‐biased agonists may be a safer strategy to treat alcoholism.

## 1 INTRODUCTION

**Figure 1:**
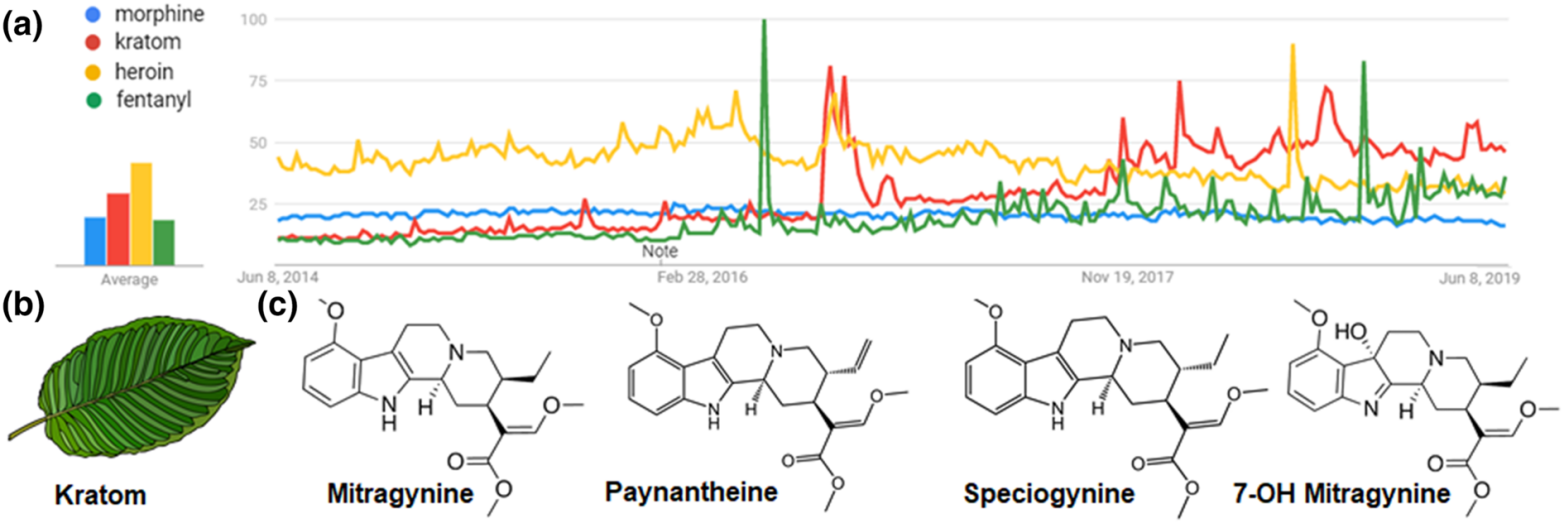
Interest in kratom has steadily increased over the last 5‐year period. Five‐year Google trends analysis from June 2014 to June 2019 (performed June 8, 2019) comparing morphine (blue), kratom (red), heroin (yellow), and fentanyl (green). Note that Google searches for kratom outnumbered those for heroin starting November 2017. The spike in heroin searches coincide with the overdose of Demi Lovato. The first spike in fentanyl searches coincide with the death of Prince and the second spike with FDA approval of Dsuvia™ (https://www.guidetopharmacology.org/GRAC/LigandDisplayForward?ligandId=3534). The initial increase in kratom searches in the fall of 2016 coincided with the DEA's decision to defer their scheduling kratom (a). Animated depiction of a kratom leaf (b). Chemical structures of characterized kratom alkaloids (c)

Interest in and use of the psychoactive plant *Mitragyna speciosa* (kratom) has risen dramatically across North America and Europe over the last 5 years (Singh, Narayanan, & Vicknasingam, 2016), (Figure 1a,b). Historically, kratom has been used in its indigenous Southeast Asian regions to relieve pain, diarrhoea and cough or to provide stimulation (Prozialeck, Jivan, & Andurkar, 2012). The currently inflated interest in kratom in the United States coincides with changes in opioid prescribing guidelines by the Center for Disease Control and Prevention in 2016 (Renthal, 2016) and the rise in https://www.guidetopharmacology.org/GRAC/LigandDisplayForward?ligandId=9082 adulterated with https://www.guidetopharmacology.org/GRAC/LigandDisplayForward?ligandId=1626‐like opioids, leading to a spike in fatal and non‐fatal overdoses (Dowell, Noonan, & Houry, 2017; Gostin, Hodge, & Noe, 2017). Kratom contains more than 40 alkaloids with varying affinity and activity at opioid receptors (Adkins, Boyer, & McCurdy, 2011; Brown, Lund, & Murch, 2017; Hassan et al., 2013; Kruegel et al., 2016; Takayama, 2004) and is commonly used for the self‐medication of opioid dependence and withdrawal, the management of chronic pain and mood disorders or as substitute for heroin or prescription opioids (Grundmann, 2017; Singh et al., 2016; Smith & Lawson, 2017). Despite these perceived benefits, increasing rates of kratom use have led to concomitant increases in reports of adverse effects following consumption, although to date, no fatal overdoses have been attributed to kratom use alone (Cinosi et al., 2015; Kruegel & Grundmann, 2017). While the Drug Enforcement Administration recently decided to withhold its decision on classifying kratom as a Schedule I drug (Griffin & Webb, 2018; Grundmann, Brown, Henningfield, Swogger, & Walsh, 2018), reservations about the safety of kratom remain, leading to increased scrutiny of its current legal status in the United States (Henningfield, Fant, & Wang, 2018; Prozialeck, 2016).

The ability of kratom alkaloids to stimulate the https://www.guidetopharmacology.org/GRAC/ObjectDisplayForward?objectId=319 (μOP) is a major factor in their activity (Angkurawaranon et al., 2018; Boyer, Babu, & Macalino, 2007; Hassan et al., 2013). Particularly, 7‐hydroxymitragynine, with (K_i_) affinities for μOP, https://www.guidetopharmacology.org/GRAC/ObjectDisplayForward?objectId=317 (δOP) and https://www.guidetopharmacology.org/GRAC/ObjectDisplayForward?objectId=318 (κOP) of 37 ± 4 nM, 91 ± 8 nM and 132 ± 7 nM, respectively (Varadi et al., 2016), has been of particular interest because of prior reports indicating 4–5× more potent antinociceptive activity than morphine in rodents while also producing less constipation (Matsumoto et al., 2004; Matsumoto et al., 2006; Takayama et al., 2002; Varadi et al., 2016). The potential reduced side‐effect profile of kratom alkaloids has been associated with their negligible μOP‐mediated β‐arrestin 2 recruitment (Kruegel et al., 2016; Varadi et al., 2016), characterizing them as so‐called G protein‐biased agonists (Majumdar & Devi, 2018; Schmid et al., 2017; Whalen, Rajagopal, & Lefkowitz, 2011).

Only a small number of pharmacological treatment options are approved for the treatment of alcohol use disorder, all of which are limited by inconsistent and/or poor efficacy. This lack of effective therapies may explain why kratom has also been reportedly used in the self‐medication of symptoms associated with alcohol withdrawal (Havemann‐Reinecke, 2011; McWhirter & Morris, 2010; Singh, Muller, & Vicknasingam, 2014; Suhaimi et al., 2016). Previous research has shown that G protein‐biased δOP agonists decrease voluntary alcohol intake in C57Bl/6 male mice, while δOP agonists that strongly recruit β‐arrestin 2 increase voluntary alcohol intake (Chiang, Sansuk, & van Rijn, 2016; Nielsen et al., 2012; Robins, Chiang, Mores, Alongkronrusmee, & van Rijn, 2018). From these findings, we hypothesized that the reported utility of kratom in reducing alcohol intake stems from kratom's constituent alkaloids displaying G protein bias at δOP. To address our hypothesis, we characterized the μOP, δOP and κOP pharmacology of two separate kratom extracts, four isolated major kratom alkaloids (mitragynine, speciogynine, paynantheine and 7‐hydroxymitragynine, Figure 1c), and three synthetic opioids *N*‐cycloheptyl‐1‐phenethyl‐4‐(*N*‐phenylpropionamido)piperidine‐4‐carboxamide, *N*‐cyclopropyl‐1‐phenethyl‐4‐(*N*‐phenylpropionamido)piperidine‐4‐carboxamide, *N*‐(tert‐butyl)‐1‐phenethyl‐4‐(*N*‐phenylpropionamido)piperidine‐4‐carboxamide (MP102, MP103 and MP105, respectively) that have G protein‐biased pharmacology similar to the kratom alkaloids. These extracts and drugs were also assessed for their ability to modulate alcohol intake, affect general locomotive behaviour,and for their rewarding properties.

## 2 METHODS

### 2.1 Materials

Speciogynine, paynantheine, 7‐hydroxymitragynine, and mitragynine were isolated (purity; >95%) by column chromatography and provided by Dr Majumdar. MP102, MP103, and MP105 were synthetically derived (purity; >97%) and provided by Dr Majumdar. https://www.guidetopharmacology.org/GRAC/LigandDisplayForward?ligandId=1627 sulfate pentahydrate, https://www.guidetopharmacology.org/GRAC/LigandDisplayForward?ligandId=1613, https://www.guidetopharmacology.org/GRAC/LigandDisplayForward?ligandId=5190, hydrochloric acid, sodium sulfate, dichloromethane, ammonia, hexanes, and ethyl alcohol (200 proof) were purchased from Sigma‐Aldrich (St. Louis, MO, USA). https://www.guidetopharmacology.org/GRAC/LigandDisplayForward?ligandId=1641 hydrochloride, (2*S*)‐2‐[[2‐[[(2*R*)‐2‐[[(2*S*)‐2‐Amino‐3‐(4‐hydroxyphenyl)propanoyl]amino]propanoyl]amino]acetyl]‐methylamino]‐*N*‐(2‐hydroxyethyl)‐3‐phenylpropanamide (https://www.guidetopharmacology.org/GRAC/LigandDisplayForward?ligandId=1647), and 2‐(3,4‐dichlorophenyl)‐*N*‐methyl‐*N*‐[(1*R*,2*R*)‐2‐pyrrolidin‐1‐ylcyclohexyl]acetamide (https://www.guidetopharmacology.org/GRAC/LigandDisplayForward?ligandId=1652) were purchased from Tocris Bioscience (Bio‐techne Corporation, Minneapolis, MN, USA). (3‐Methoxythiophen‐2‐yl)methyl]((2‐[(9*R*)‐9‐(pyridin‐2‐yl)‐6‐oxaspiro‐[4.5]decan‐9‐yl]ethyl))amine (TRV130, https://www.guidetopharmacology.org/GRAC/LigandDisplayForward?ligandId=7334) was purchased from AdooQ Bioscience (Irvine, CA, USA). For animal drinking assays, pure ethyl alcohol was diluted to 10% or 20% alcohol in reverse osmosis water.

### 2.2 Kratom extract #1

An alkaloid extract was obtained from Maeng Da Micro Powder (MoonKratom, Austin, TX, USA) as described previously by Orio, Alexandru, Cravotto, Mantegna and Barge (2012). In brief, as shown in Figure S1A, extraction was performed by treating kratom powder in 95% ethanol at 50°C for 4 hr followed by removal of the remaining organic material by vacuum filtration. Solvent was then removed under reduced pressure and the crude extract resuspended in dilute aqueous hydrochloric acid (pH = 3) and washed with hexane. The aqueous solution was then basified (pH = 9) with 0.1‐M aqueous ammonia and the freebase alkaloid fraction extracted with dichloromethane. The alkaloid containing fraction was dried over anhydrous sodium sulfate and filtered, followed by removal of solvent under reduced pressure and further drying under high vacuum to obtain our kratom alkaloid extract as a crystalline, light brown solid. Extract composition was assessed on a 6550‐quadropole time of flight (Aligent, Santa Clara, CA, USA; scan 105–100 amu) using a Zorbax Extend‐C18 column (Aligent) held at 30°C and a 0.3 ml·min^−1^ of flow rate.

### 2.3 Kratom extract #2

Mitragynine was extracted from the powdered leaves by following our previously reported methods (Varadi et al., 2016; Figure S1B). “Red Indonesian Micro Powder” was purchased from MoonKratom. The kratom powder (500 g) was heated to 75°C to reflux in methanol 700 ml for 40 min. The suspension was filtered, and the methanolic extraction process was repeated (3 × 500 ml). The solvent of combined methanolic extract was removed under reduced pressure, and the content was dried using high vacuum. The dry residue was resuspended in 20% acetic acid solution (1 L; pH = 4) and washed with petroleum ether (4 × 500 ml). The aqueous layer was then cooled on ice bath and basified (pH ~9) slowly with aqueous sodium hyrdoxide solution (3.5 M; ~1 L). Alkaloids were extracted in dichloromethane (4 × 400 ml) from the aqueous layer. The combined dichloromethane fractions were washed with brine (300 ml) and dried over anhydrous sodium sulfate and filtered. The solvent was removed under reduced pressure, and the residue was dried under high vacuum to obtain kratom extract. The kratom extract was subjected to column chromatography (gradient: 0–40% ethylacetate in hexanes) to isolate mitragynine (Yield; 4.9 g), and smaller quantities of paynantheine (0.58 g) and speciogynine (0.35 g).

For cellular assays, kratom extracts were dissolved to a concentration of 10 mM in 100% DMSO. The calculated concentration was estimated by assigning the kratom extract an estimated molecular mass of 400 g·mol^−1^, which is the average size of kratom alkaloids.

### 2.4 Cell culture and biased signalling assays

cAMP inhibition and β‐arrestin 2 recruitment assays were performed as previously described (Chiang et al., 2016). In brief, for cAMP inhibition assays, HEK 293 (RRID:CVCL_0045, Life Technologies, Grand Island, NY, USA) cells were transiently transfected in a 1:3 ratio with FLAG‐mouse δOP, HA‐mouse μOP, or FLAG‐mouse κOP and pGloSensor22F‐cAMP plasmids (Promega, Madison, WI, USA) using Xtremegene9 (Sigma). Two days post‐transfection, cells (20,000 cells per well, 7.5 μl) were seeded in low‐volume Greiner 384‐well plates (#82051‐458, VWR, Batavia, IL, USA) and incubated with GloSensor reagent (Promega, 7.5 μl, 2% final concentration) for 90 min at room temperature. Cells were stimulated with 5‐μl drug solution for 20 min at room temperature prior to stimulation with 5‐μl forskolin (final concentration 30 μM) for an additional 15 min at room temperature. For β‐arrestin recruitment assays, CHO‐K1‐human μOP PathHunter β‐arrestin 2 cells (RRID:CVCL_KY70) and CHO‐K1‐human δOP PathHunter β‐arrestin 2 cells (RRID:CVCL_KY68) or U‐2 osteosarcoma (U2OS)‐human κOP PathHunter β‐arrestin 2 cells (RRID:CVCL_LA97, DiscoverX, Fremont, CA, USA) were plated (2,500 cells per well, 10 μl) 1 day prior to stimulation with 2.5‐μl drug solution for 90 min at 37°C/5%CO_2,_ after which cells were incubated with 6‐μl cell PathHunter assay buffer (DiscoverX) for 60 min at room temperature as per the manufacturer's protocol. Luminescence for each of these assays was measured using a FlexStation3 plate reader (Molecular Devices, Sunnyvale, CA, USA).

### 2.5 Calculation of bias factor

In order to determine ligand bias, we followed the operational model equation in Graphpad Prism 8 (RRID:SCR_002798, GraphPad Software, La Jolla, CA) to calculate Log R (τ/KA; Table S1), ΔLogR, and ΔΔLogR as previously described (van der Westhuizen, Breton, Christopoulos, & Bouvier, 2014). Subsequently, bias factors (10^ΔΔLogR^) were calculated using DAMGO, leu‐enkephalin, and U50,488 as reference compounds for μOP, δOP, and κOP, respectively. All three reference compounds were more potent in the cAMP (G protein) assay than in the β‐arrestin 2 recruitment assay and thus were not unbiased but G protein‐biased to begin with. A bias factor >1 meant that the agonist was more G protein‐biased than the reference compound; a bias factor <1 meant that the agonist was less G protein‐biased than the reference compound. Bias factors for compounds with <30% efficacy for β‐arrestin 2 recruitment could not reliably be calculated and are listed as undeterminable (Table 1), which indicates that these agonists can be considered to be efficacy dominant for G protein signalling (Kenakin, 2015).

**Table 1:** Pharmacological characterization of kratom alkaloids and synthetic G protein‐biased opioids at the μ, δ and κ opioid receptor

	cAMP		β‐arrestin 2		
Compounds	pIC_50_	*α*	pEC_50_	*α*	Bias factor
μOR					
DAMGO	8.4 ± 0.1 (23)	100	6.7 ± 0.1 (22)	100	1
Kratom #1	6.0 ± 0.2 (7)	71 ± 7	ND (3)	ND	UD
Kratom #2	5.7 ± 0.3 (3)	100 ± 7	ND (3)	ND	UD
Morphine	8.5 ± 0.2 (3)	95 ± 4	6.8 ± 0.1 (3)	26 ± 2	UD
Mitragynine	6.3 ± 0.2 (8)	75 ± 6	ND (3)	ND	UD
7‐OH‐mitragynine	7.8 ± 0.1 (5)	84 ± 3	ND (3)	ND	UD
Speciogynine	5.5 ± 0.1 (5)	87 ± 6	ND (3)	ND	UD
Paynantheine	5.4 ± 0.1 (5)	100 ± 0	ND (3)	ND	UD
TRV130	7.9 ± 0.2 (7)	86 ± 3	ND (4)	ND	UD
MP102	5.4 ± 0.2 (6)	88 ± 4	5.2 ± 0. 1(4)	16 ± 5	UD
MP103	6.5 ± 0.2 (5)	90 ± 4	6.3 ± 0.2 (7)	63 ± 7	0.03
MP105	6.7 ± 0.4 (5)	87 ± 6	6.6 ± 0.2(6)	54 ± 5	0.02
δOR					
Leu‐enkephalin	8.5 ± 0.1 (34)	100	8.0 ± 0.1 (29)	100	1
Kratom #1	4.4 ± 0.3 (6)	30 ± 20	ND (4)	ND	UD
Kratom #2	5.8 ± 0.5 (4)	72 ± 13	ND (3)	ND	UD
Morphine	6.1 ± 0.2 (5)	82 ± 8	ND (3)	ND	UD
Mitragynine	4.8 ± 0.2 (5)	88 ± 8	ND (3)	ND	UD
7‐OH‐mitragynine	5.7 ± 0.2 (8)	80 ± 8	6.4 ± 0.3 (3)	14 ± 1	UD
Speciogynine	5.0 ± 0.3 (5)	94 ± 4	ND (3)	ND	UD
Paynantheine	5.6 ± 0.2 (4)	64 ± 13	ND (3)	ND	UD
TRV130	5.6 ± 0.4 (5)	48 ± 12	ND (4)	ND	UD
MP102	6.4 ± 0.1 (4)	77 ± 7	6.7 ± 0.4 (5)	20 ± 6	UD
MP103	5.5 ± 0.2 (4)	100 ± 1	5.8 ± 0.1 (3)	35 ± 2	3.8
MP105	5.4 ± 0.3 (4)	94 ± 5	6.2 ± 0.1 (3)	35 ± 4	1.4
κOR					
U50,488	8.9 ± 0.1 (18)	100	7.3 ± 0.2 (10)	100	1
Kratom #1	6.8 ± 0.4 (8)	41 ± 8	ND (6)	ND	UD
Kratom #2	7.0 ± 0.2 (4)	93 ± 2	6.0 ± 0.2 (5)	12 ± 2	UD
Morphine	7.3 ± 0.2 (5)	89 ± 2	5.8 ± 0.2 (3)	16 ± 2	UD
Mitragynine	5.4 ± 0.4 (5)	67 ± 12	ND (4)	ND	UD
7‐OH‐mitragynine	6.2 ± 0.3 (9)	77 ± 5	ND (4)	ND	UD
Speciogynine	4.7 ± 0.3 (5)	70 ± 20	ND (4)	ND	UD
Paynantheine	5.3 ± 0.2 (4)	95 ± 5	ND (6)	ND	UD
TRV130	4.9 ± 0.1 (3)	74 ± 6	ND (3)	ND	UD
MP102	5.4 ± 0.1 (5)	85 ± 8	ND (3)	ND	UD
MP103	6.0 ± 0.1 (3)	93 ± 3	ND (3)	ND	UD
MP105	5.2 ± 0.2 (5)	96 ± 3	ND (3)	ND	UD

*Note.* cAMP inhibition potencies (pIC_50,_ drug concentration at 50% maximal efficacy) and efficacies (*α*, % inhibition at maximal efficacy normalized to DAMGO [μOP], leu‐enkephalin [δOP], or U50,488 [κOP]) for OP agonists to inhibit cAMP production are indicated ±SEM. β‐arrestin 2 recruitment potencies (pEC_50_) and efficacies (*α*, normalized to DAMGO, leu‐enkephalin, or U50,488) of OP agonists to recruit β‐arrestin 2 are indicated ±SEM. The number of repetitions for each drug is indicated in parentheses. ND, not detectable. For Log R (τ/KA) values with confidence intervals, see Table S1.

### 2.6 Animals

The animal protocol (#1305000864) describing the care and use of experimental animals was approved by the Purdue University Institutional Animal Care and Use Committee (https://www.purdue.edu/research/regulatory-affairs/animal-research/staff.php). Animal studies are reported in compliance with the ARRIVE guidelines (Kilkenny et al., 2010) and with the recommendations made by the *British Journal of Pharmacology* as well as recommendations of the National Institutes of Health Guide for the Care and Use of Laboratory Animals. For our experiments, we used adult male (19–24 g) and female (17–21 g) wild‐type C57/BL6NHsd mice (8–10 weeks old) purchased from Envigo (#044, Indianapolis, IN, USA) but originated from the National Institutes of Health. This is a strain known to readily consume alcohol (Belknap, Crabbe, & Young, 1993). We also used male δOP knockout C57BL/6 mice of similar age in a subset of experiments. δOP knockout mice were produced by removal of exon 2 as previously described (van Rijn & Whistler, 2009) and outbred to a C57BL/6 background (>10 generations). Roughly every 3 years, the strain is backcrossed to commercially obtained C57BL/6 mice (Envigo) to mitigate the effects of genetic drift.

We provided food and water ad libitum unless specified otherwise for the binge ethanol experiments. With the exception of the ethanol experiments where mice were individually housed in double grommet cages, animals were group housed in plexiglass cages in ventilated racks at ambient temperature of (21°C) in a room maintained on a reversed 12L:12D cycle (lights off at 10.00, lights on at 22.00) in Purdue University's animal facility, which is accredited by the Association for Assessment and Accreditation of Laboratory Animal Care. Mice were used only in a single behavioural paradigm with the exception of the two‐bottle choice model of moderate 10% alcohol consumption (Section 2.6) in which mice received increasing doses of the test drug over multiple weeks.

### 2.7 Two‐bottle choice model of moderate 10% alcohol consumption

Mice were trained to voluntarily consume alcohol in a limited access (4 hr·day^−1^), 2‐bottle choice (water vs. 10% ethanol), drinking‐in‐the‐dark protocol during their active phase (3 hr after the start of the dark cycle) until the alcohol intake was stable as previously described (van Rijn & Whistler, 2009). During the first 3 weeks of limited alcohol access, the mice increased their alcohol intake prior to reaching steady state consumption. After the completion of the third week of voluntary alcohol intake, injections were administered every Friday 30 min prior to the 4‐hr drinking session. Drug effect on alcohol and water intake was measured as a change in Friday total drinking minus average alcohol intake between Tuesday–Thursday (g·kg^−1^). Raw data for alcohol intake, water intake, alcohol preference and associated statistical analysis are provided in Figures S2–S4 and Table S2.

### 2.8 Intermittent, limited access of 20% alcohol binge consumption model

To measure the effects of drug administration on binge‐like alcohol consumption, we followed a drinking‐in‐the‐dark binge protocol (Rhodes, Best, Belknap, Finn, & Crabbe, 2005; Robins et al., 2018). In this 1‐week protocol, the water bottle for each cage was replaced with a bottle containing 20% ethanol for 2 hr on Monday–Thursday (i.e. no 2‐bottle choice). Using consumption data from these 4 days, the average ethanol consumption of the mice was ranked, and the mice were sorted into groups of comparable/equal drinking (with each group to be administered either vehicle or a drug). On the Friday of the binge protocol, mice received IP injections of either vehicle or drug 30 min prior to a 4‐hr “binge” drinking period with access to 20% ethanol. To determine drug effect on alcohol intake, the amount of ethanol consumed during the binge period was compared between vehicle and drug‐treated groups.

For all ethanol consumption experiments, bottle weights were measured directly before and after the ethanol access periods to the second decimal point to determine fluid intake and weights of bottles were corrected for any spillage. In addition, the location of alcohol bottles in each paradigm was alternated daily (right vs. left grommet) to prevent habit formation.

### 2.9 General locomotor activity assessment

Square locomotor boxes from Med Associates (L 27.3 cm × W 27.3 cm × H 20.3 cm, St. Albans, VT, USA) were used to monitor locomotor activity. For all locomotor studies, animals were moved to the testing room for 60 min prior to testing for habituation. A 90‐min baseline habituation session to the boxes was conducted prior to drug administration to reduce novelty locomotor differences. The following day, mice were again habituated to the room for 60 min. Then mice were injected with drug or vehicle, and locomotor activity was monitored immediately for a total of 90 min. All testing was conducted during the dark/active phase.

### 2.10 Acute thermal antinociception

To measure antinociception, we utilized a tail‐flick assay as previously described (van Rijn, Brissett, & Whistler, 2012b). In short, 8‐week‐old male C57BL/6 wild‐type mice (*n* = 10) were habituated to handling. The following day a baseline tail‐flick response was recorded using a radiant‐heat tail‐flick apparatus (Columbus Instruments, Columbus, OH, USA). The light intensity was set to “9” to produce an average baseline response of 2–3 s. We utilized a maximal cut‐off of 3× baseline to reduce the risk of damaging the mice tails. For each test, two tail‐flick responses were recorded, and the average was used for further analysis. Immediately after the baseline recording, mice were injected s.c. with saline and 30 min later, a new tail‐flick response was measured. After the saline injection, mice were injected with 1 mg·kg^−1^ of TRV130 (s.c.), and a third tail‐flick response was recorded 30 min post‐TRV130 injection. The experiment was repeated in the same cohort of mice on the following 2 days but using 3 and 10 mg·kg^−1^ of TRV130 instead. We only tested eight mice with 10 mg·kg^−1^ of TRV130 and injected the remaining two mice with 10 mg·kg^−1^ of morphine as internal control (both mice displayed 100% antinociception, data not shown). Antinociception was calculated as maximal possible effect (%MPE) = (Response_drug_ − Response_baseline_)/(Response_cut‐off_ − Response_baseline_) * 100.

### 2.11 “Brief” conditioned place preference

Mice were conditioned to drugs or vehicle as described previously (Varadi, Marrone, et al., 2015) with two modifications: (a) conditioning sessions lasted 40 min rather than 30 min and (b) a two‐chamber apparatus rather than a three‐chamber set‐up was utilized. One chamber contained a wire mesh floor and horizontal black/white striped wallpaper, whereas the second chamber contained a metal rod floor and vertical black/white striped wallpaper. To determine initial compartment bias, a vehicle injection was administered i.p. immediately prior to the pre‐conditioning session to create an unbiased, counterbalanced approach for drug pairing (half of the animals received drug on the pre‐test preferred side, while half received drug on the pre‐test non‐preferred side). Animals exhibiting >70% preference for one of the two chambers were removed from further testing. Over the following 2 days, two conditioning sessions per day were performed 4 hr apart (morning and afternoon, vehicle, or drug semi‐random) for a total of four conditioning sessions (two to vehicle on the non‐drug‐paired side and two to drug on the drug‐paired side). On the post‐conditioning testing day, a vehicle injection was administered directly before placing the animals in the testing apparatus to determine post‐conditioning preference. For all sessions, animals were habituated to the testing room 60 min before sessions, and all behaviour was conducted during the dark/active phase.

### 2.12 “Extended” conditioned place preference

The differences between the “brief” and “extended” conditioned place preference (CPP) were as follows: (a) Conditioning sessions were 30 min instead of 40 min, (b) mice only received one conditioning session per day (none on the weekend) and (c) mice received four rather than two vehicle and drug exposures. In the initial habituation session, a vehicle i.p. injection was administered prior to the session to assess initial bias towards either chamber. An unbiased, counterbalanced approach was then used to assign the drug‐paired side for each animal (half of the animals received drug on the pre‐test preferred side, while half received drug on the pre‐test non‐preferred side). Animals exhibiting >70% preference for one of the two chambers were removed from further testing. Over the course of 2 weeks, mice were conditioned on 8 days, but with only one conditioning session per day (four to vehicle on the non‐drug‐paired side and four to drug on the drug‐paired side). On the post‐conditioning testing day, a vehicle injection was administered directly before placing the animals in the testing apparatus where animals were allowed to explore both chambers to determine post‐conditioning preference. For all sessions, animals were habituated to the testing room 60 min before sessions, and all behaviour was conducted during the dark/active phase.

### 2.13 Data and statistical analysis

The data and statistical analysis comply with the recommendations of the *British Journal of Pharmacology* on experimental design and analysis in pharmacology (Curtis et al., 2018). All data are presented as means ± SEM, and analysis was performed using GraphPad Prism 8 software. For in vitro assays, non‐linear regression was conducted to determine pIC_50_ (cAMP) or pEC_50_ (β‐arrestin 2 recruitment). Technical replicates were used to ensure the reliability of single values, specifically each data point for binding, and arrestin recruitment was run in duplicate, and for the cAMP assay in triplicate. The averages of each independent run were considered a single experiment and combined to provide a composite curve in favour of providing a “representative” curve. In each experimental run, a positive control/standard was utilized to allow the data to be normalized, thereby providing the opportunity to calculate the log bias value which relies on the presence of the standard. For the data analysis of the behavioural experiments, we first we established that data set did not contain an outlier using the Grubbs' test. If the test revealed an outlier, this value was removed, but removal was limited to one data point per set. We then verified if the data values came from a Gaussian distribution using the D'Augostino and Pearson omnibus normality test. If the data followed a Gaussian distribution, we carried out a parametric test; otherwise, we opted for the non‐parametric test. To determine statistical differences in the means between two values, we performed a Student's unpaired *t‐*test if the two datasets passed the normality test, but with a Welch's correction if the datasets did not have the same SD. For those datasets, where one or both datasets did not pass the normality test, we performed a Mann–Whitney *U* test. Significant changes in average alcohol intake were determined by one‐way, repeated measures ANOVA with Tukey's multiple comparisons (MC) test. For CPP, two‐way, repeated measures ANOVA with Bonferroni MC was used to determine significant differences in time spent on the drug‐paired side pre‐ versus post‐conditioning. One‐way ANOVA with Bonferroni MC determined significance for locomotor studies. For the repeated measures tests, whenever we could not assume sphericity, a Geisser–Greenhouse correction was carried out by GraphPad Prism 8 software. Post hoc tests were conducted only if F in ANOVA achieved P < .05 and there was no significant variance inhomogeneity. In this study, *P* values <.05 were considered as statistically significant (Tables S3–S6). Whenever possible, the experimenter was blind to the drug and/or dose tested; however, we always started with the lowest drug dose if multiple doses were to be tested. Animals were assigned to groups such that the baseline responding was equal across groups. The treatment that each group received was then randomized. Group sizes were equal by design and based on a power analysis calculated using the observed deviation in our prior published work. On occasion, mice were excluded prior to drug treatment because of a failure to consume alcohol during the initial alcohol voluntary consumption phase; however, on some occasions, we started with a larger group size to account for potential non‐responders. We did not explicitly design our experiments to test for sex differences; with the exception of the study of kratom impact on binge alcohol use, male and female groups were of unequal size. To account for the unequal sample size, we utilized the Sidak's post hoc test.

### 2.14 Nomenclature of targets and ligands

Key protein targets and ligands in this article are hyperlinked to corresponding entries in http://www.guidetopharmacology.org, the common portal for data from the IUPHAR/BPS Guide to PHARMACOLOGY (Harding et al., 2018), and are permanently archived in the Concise Guide to PHARMACOLOGY 2019/20 (Alexander et al., 2019).

## 3 RESULTS

### 3.1 Different strains of kratom differ slightly in alkaloid composition

The Maeng Da kratom extract #1 consisted of mitragynine (44.9%), paynantheine (14.1%), speciogynine (8.9%) and 7‐hydroxymitragynine (2.9%) as the major alkaloids. In contrast, Red Indonesian Micro powder kratom extract #2 was notably devoid of 7‐hydroxymitragynine and consisted of mitragynine (49%), paynantheine (9%) and speciogynine (3.5%). The alkaloid compositions of the kratom extracts were in line with those previously reported (Hassan et al., 2013).

### 3.2 Mice injected with kratom extract decrease moderate and binge alcohol intake

**Figure 2:**
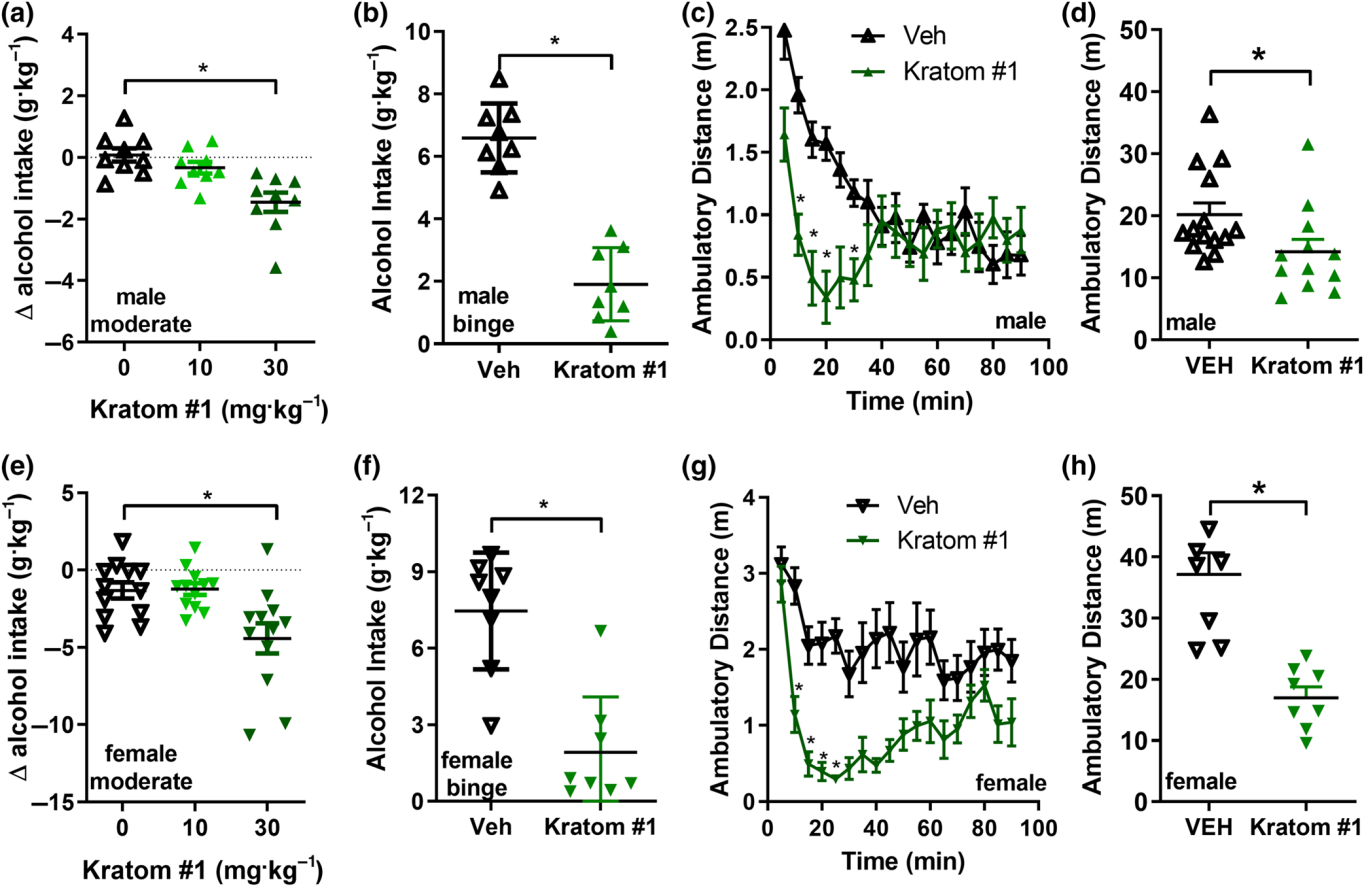
Kratom reduces alcohol intake and locomotor activity in male and female mice. Systemic (i.p.) injection of kratom extract #1 dose‐dependently reduced 10% alcohol intake in male mice (a). Systemic administration of 30 mg·kg^−1^ of kratom extract #1 decreased 20% alcohol intake in male mice (b). Time course of male locomotor activity following kratom extract #1 injection (30 mg·kg^−1^, i.p.) (c). Systemic administration of 30 mg·kg^−1^ of kratom extract #1 reduced general locomotor activity of male mice (d). Systemic (i.p.) injection of kratom extract #1 dose‐dependently reduced 10% alcohol intake in female mice (e). Systemic administration of 30 mg·kg^−1^ of kratom extract #1 decreased 20% alcohol intake in female mice (f). Time course of female locomotor activity following kratom extract #1 injection (30 mg·kg^−1^, i.p.) (g). Systemic administration of 30 mg·kg^−1^ of kratom extract #1 reduced general locomotor activity of female mice (h). See Section 3 for statistical analysis

To corroborate the self‐prescribed use of kratom for alcohol use disorder, male C57BL/6 mice (*n* = 9) were injected (i.p.) with kratom extract #1, resulting in a dose‐dependent decrease in their volitional alcohol intake (Figure 2a), with the dose of 30 mg·kg^−1^ of kratom extract #1 displaying a significant decrease compared with vehicle (0.9% saline) or 10 mg·kg^−1^. We obtained a significant similar result in male mice (*n* = 10) when injected with kratom extract #2 (Figure S5). It should be noted that while kratom extract #2 did not contain 7‐hydroxymitragynine, mitragynine can be metabolized into 7‐hydroxymitragynine (Kruegel et al., 2019) and thus extracts #1 and #2 may not differ much *in vivo.* We found that 30 mg·kg^−1^ of kratom extract #1 significantly reduced alcohol intake in male C57BL/5 mice (*n* = 8) in a model of 20% alcohol binge use (Figure 2b). We assessed if mice receiving 30 mg kg^−1^ of kratom extract #1 displayed altered locomotor activity compared with vehicle‐treated male mice (vehicle, *n* = 14; kratom, *n* = 12). Over a 90‐min timespan, mice receiving kratom extract #1 had a slight but significant decrease in their total locomotor activity compared with vehicle‐treated mice (Figure 2c). The difference was driven by a rapid but short decrease in locomotor activity in the first 30 min following injection (Figure 2d). As for the male mice, we also observed that kratom extract #1 dose‐dependently decreased alcohol intake in female mice (Figure 2e, *n* = 12) and 30 mg·kg^−1^ of kratom extract #1 similarly decreased binge alcohol intake in female mice (Figure 2f, *n* = 8). The dose of 30 mg·kg^−1^ of kratom extract #1 decreased locomotor activity in female mice (Figure 2g, *n* = 8). As for the males, a sharp decrease in locomotor activity was observed during the first 10–40 min post‐injection (Figure 2h). Sex differences exist for basal alcohol intake by C57BL/6 mice (Nocjar, Middaugh, & Tavernetti, 1999; Robins et al., 2018) and were apparent for moderate alcohol intake 3.0 versus 5.0 mg·kg^−1^, but not significant for binge alcohol intake 6.6 versus 7.5 mg·kg^−1^. There was a sex–drug interaction for the locomotor effects of kratom, but not for modulation of moderate alcohol intake or binge alcohol intake.

### 3.3 Kratom alkaloid extracts exhibit G protein bias

Both kratom extracts inhibited forskolin‐stimulated cAMP production in HEK293 cells transiently transfected with mouse μOP, δOP, or κOP, although with decreased potency and efficacy compared with the reference ligands DAMGO, leu‐enkephalin, and U50,488 respectively. In general, the kratom extracts were less potent than morphine, with the exception of kratom extract #2 at κOP. The reference opioids all efficaciously recruited β‐arrestin 2 while morphine weakly recruited β‐arrestin 2 at μOP. The kratom extracts did not lead to detectable β‐arrestin 2 recruitment at μOP or δOP, with very weak recruitment at κOP, similar to that of morphine at κOP (Table 1 and Figure S6).

### 3.4 Kratom alkaloids display G protein bias at all three opioid receptors in vitro

We next assessed if the individual kratom alkaloids mitragynine, paynantheine, speciogynine, and 7‐hydroxymitragynine (Figure 1c) are G protein‐biased at all three opioid receptors by measuring cAMP inhibition and β‐arrestin 2 recruitment at μOP, δOP, and κOPs. All four alkaloids inhibited cAMP production, with 7‐hydroxymitragynine being the most potent at all three opioid receptors. In line with the limited β‐arrestin 2 recruitment observed for the kratom extracts, the individual alkaloids did not recruit β‐arrestin 2 to a measurable extent (Table 1 and Figure S7).

### 3.5 Kratom alkaloids decrease alcohol intake but differentially affect general locomotion

**Figure 3:**
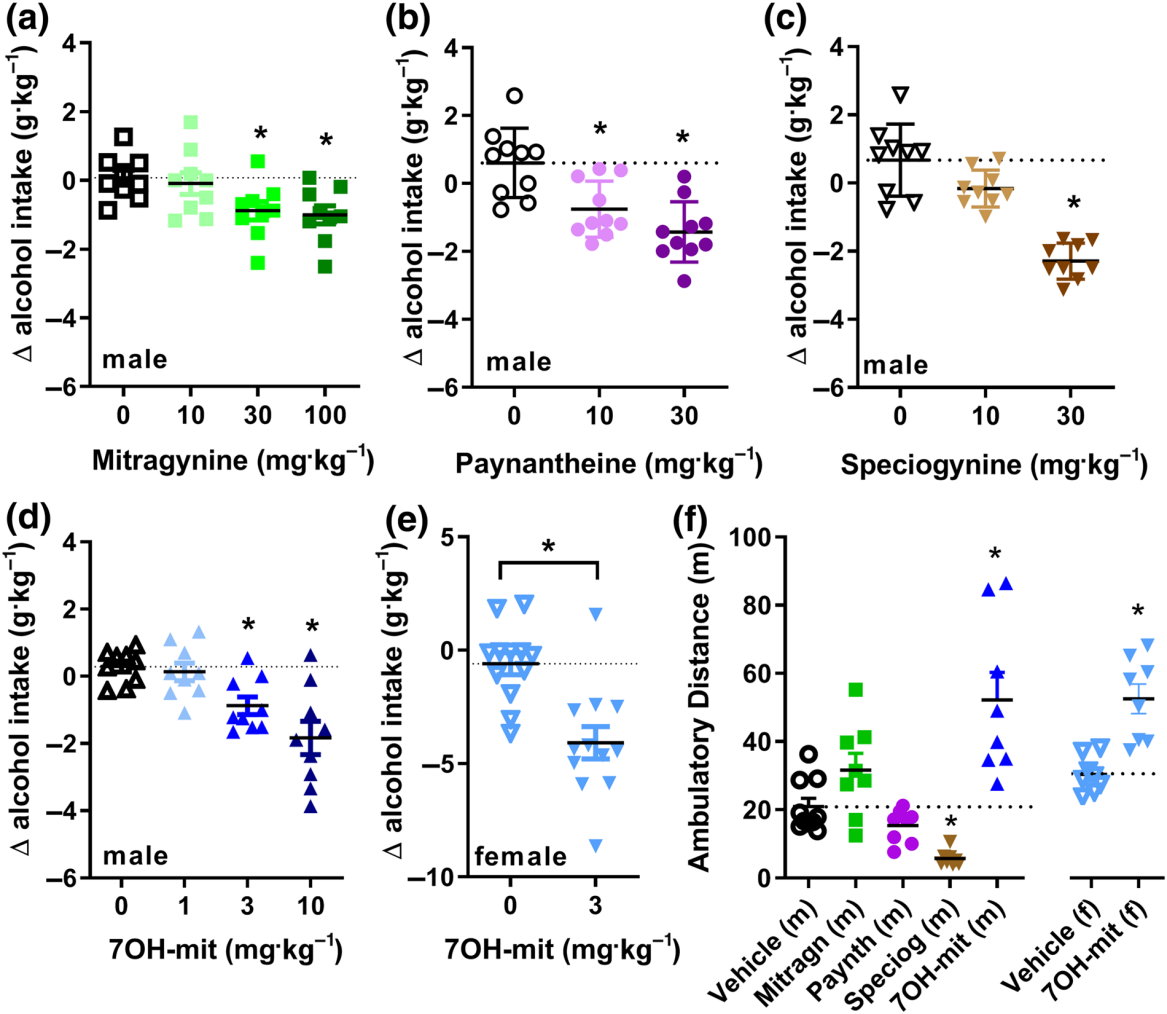
Kratom alkaloids reduce alcohol intake but differentially affect locomotor activity. Systemic (i.p.) injection of mitragynine (a), paynantheine (b), speciogynine (c), and 7‐hydroxymitragynine (7‐OH‐mit; d), on modified 2‐bottle choice drinking behaviour in male C57BL/6 mice. Systemic injection of 7‐hydroxymitragynine (3 mg·kg^−1^, i.p.), reduced 10% alcohol intake in female mice (e). 7‐hydroxymitragynine (3 mg·kg^−1^, i.p.) increased locomotor activity of male and female mice, whereas speciogynine (30 mg·kg^−1^, i.p.) decreased locomotor activity in male mice (f). See Section 3 for statistical analysis

We next investigated whether the individual kratom alkaloids would modulate alcohol intake. We chose to use male mice, as their locomotor activity was impacted less by kratom than female mice. Alcohol intake in male C57BL/7 mice was significantly decreased upon administration of 30 and 100 mg·kg^−1^ of mitragynine compared with average change in alcohol intake after vehicle (0.9% saline) injection (Figure 3a). Paynantheine dose‐dependently decreased voluntary alcohol intake at both 10 and 30 mg·kg^−1^ (Figure 3b), while speciogynine decreased alcohol intake only at the 30 mg·kg^−1^ of dose (Figure 3c). For 7‐hydroxymitragynine, the most potent of the alkaloids, a decrease in alcohol intake was observed at both 3 and 10 mg·kg^−1^ with a dose‐dependent efficacy (Figure 3d). In female mice, administration of 3 mg·kg^−1^ of 7‐hydroxymitragynine also significantly decreased alcohol intake in the same 10% voluntary ethanol consumption protocol (Figure 3e). Given that the kratom extract altered general locomotor, we also assessed whether the individual alkaloids had similar effects on locomotor activity of mice when given at the lowest effective dose. The kratom alkaloids produced variable locomotor effects in the male mice. Both 30 mg·kg^−1^ of mitragynine (*n* = 8) and 3 mg·kg^−1^ of 7‐hydroxymitragynine (*n* = 8) increased locomotor activity, although only the increase induced by 7‐hydroxymitragynine was significant compared with vehicle. In contrast, 10 mg·kg^−1^ of paynantheine (*n* = 8) did not significantly alter locomotion whereas 30 mg·kg^−1^ of speciogynine (*n* = 8) significantly decreased locomotion compared with vehicle (0.9% saline, *n* = 10) control (Figure 3f). The 3 mg·kg^−1^ dose of 7‐hydroxymitragynine also increased locomotor activity in female mice (Figure 3f).

### 3.6 Selective G protein activation of μOP is insufficient to reduce alcohol intake

**Figure 4:**
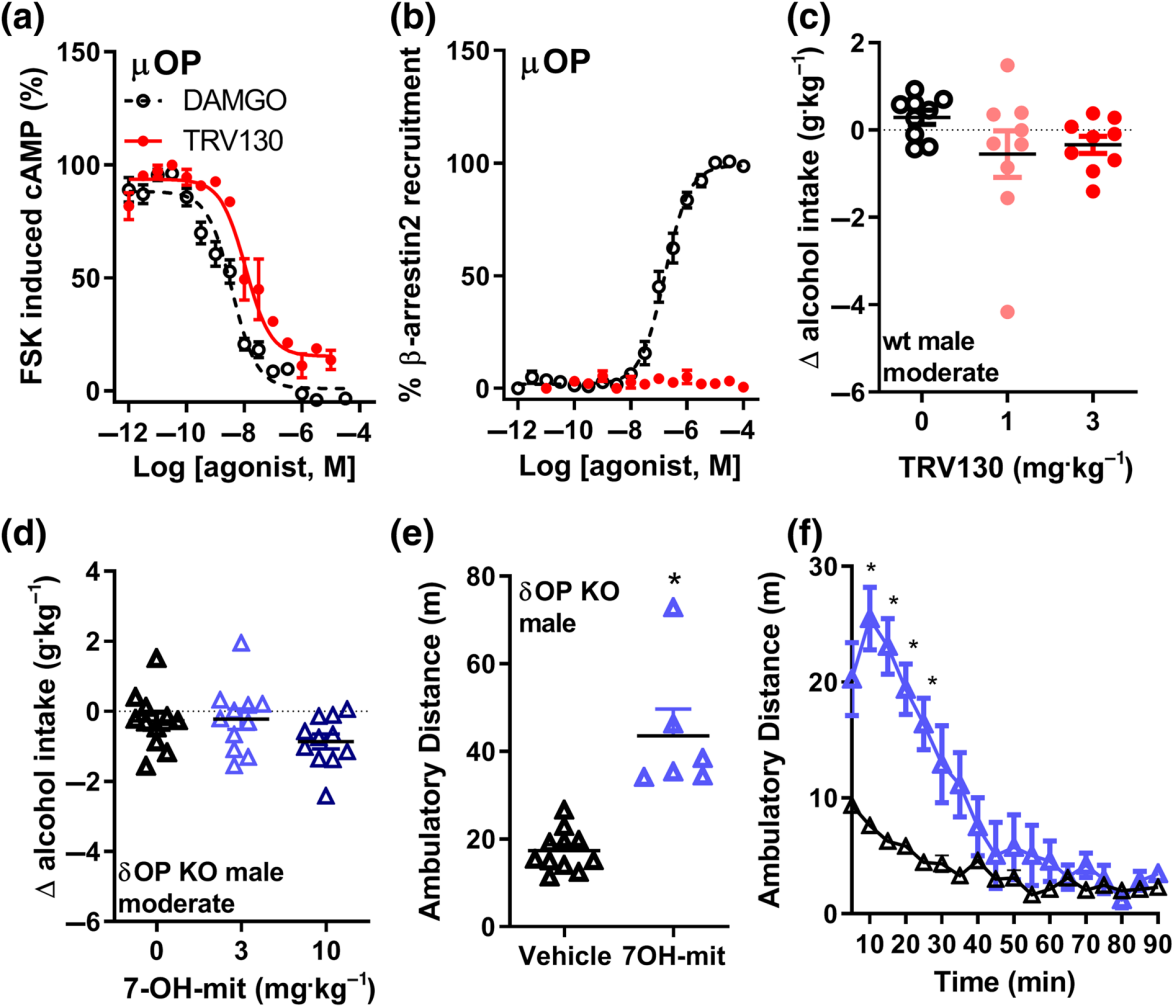
Kratom alkaloids reduce alcohol intake in mice through a δOP‐dependent mechanism. The G protein‐biased μOP agonist TRV130 (red ●) potently reduced forskolin (FSK)‐induced cAMP production in HEK293 cells expressing μOP (a, data are normalized to the positive control DAMGO, dotted line). TRV130 (red ●) did not recruit β‐arrestin 2 in CHO‐μOP (b, data are normalized to the positive control DAMGO, dotted line). Systemic (i.p.) injection of TRV130 did not decrease 10% alcohol intake in male wild‐type (WT) mice (c). Systemic (i.p.) injection of 7‐hydroxymitragynine did not decrease 10% alcohol intake in male δOP knockout (KO) male mice (d). Systemic injection of 7‐hydroxymitragynine (3 mg·kg^−1^, i.p.) increased locomotor activity of δOP knockout (KO) male mice (e). Time course of male locomotor activity following 3 mg·kg^−1^, 7‐hydroxymitragynine i.p. injection (f). See Section 3 for statistical analysis

We next sought to determine whether the changes in alcohol intake by kratom and kratom alkaloids were μOP mediated. We were limited in our approach as removal of functional μOPs through antagonism or knockout cause animals to self‐administer less alcohol (Middaugh & Bandy, 2000; Roberts et al., 2000). As an alternative approach, to determine if G protein‐biased μOP signalling underlies the observed alcohol phenotype of kratom, we investigated whether TRV130 (oliceridine), a known μOP G protein‐biased agonist (DeWire et al., 2013), could reduce alcohol intake. We first confirmed that TRV130 is a G protein‐biased and μOP‐selective agonist (Figure 4a,b, Table 1, and Figure S8). Yet, at doses (1 and 3 mg·kg^−1^, i.p.) known to produce μOP‐mediated analgesia (DeWire et al., 2013; Figure S9), TRV130 failed to decrease 10% alcohol intake in wild‐type male mice (Figure 4c) compared to vehicle (saline 0.9%).

### 3.7 7‐hydroxymitragynine's effect on alcohol intake, but not on locomotion, requires δOPs

We next examined the contribution of δOPs to these observed behaviours, particularly because G protein‐biased δOP agonists can reduce moderate alcohol intake in mice (Chiang et al., 2016; Robins et al., 2018). Because 7‐hydroxymitragynine has affinity and potency at δOPs, we assessed this alkaloids response in δOP knockout male mice. We did not observe changes in 10% alcohol intake at 3 or 10 mg·kg^−1^ of 7‐hydroxymitragynine compared to vehicle (0.9% saline) in δOP knockout mice (Figure 4d). In a different cohort of δOP knockout mice, 3 mg·kg^−1^ of 7‐hydroxymitragynine (*n* = 6) significantly increased locomotor activity compared with controls (*n* = 12; Figure 4e), with a peak in activity immediately after injection (Figure 4f). The lack of impact of 7‐hydroxymitragynine on alcohol intake in δOP knockout mice, despite increased locomotor activity, suggests that hyperlocomotion alone did not contribute to the observed decrease in alcohol intake at 3 mg·kg^−1^ of 7‐hydroxymitragynine in wild‐type mice (Figure 3d). There was no sex–drug interaction for the locomotor effects of 7‐hydroxymitragynine, but there was an interaction for modulation of moderate alcohol intake.

### 3.8 Kratom and 7‐hydroxymitragynine require more conditioning sessions to establish CPP than morphine

**Figure 5:**
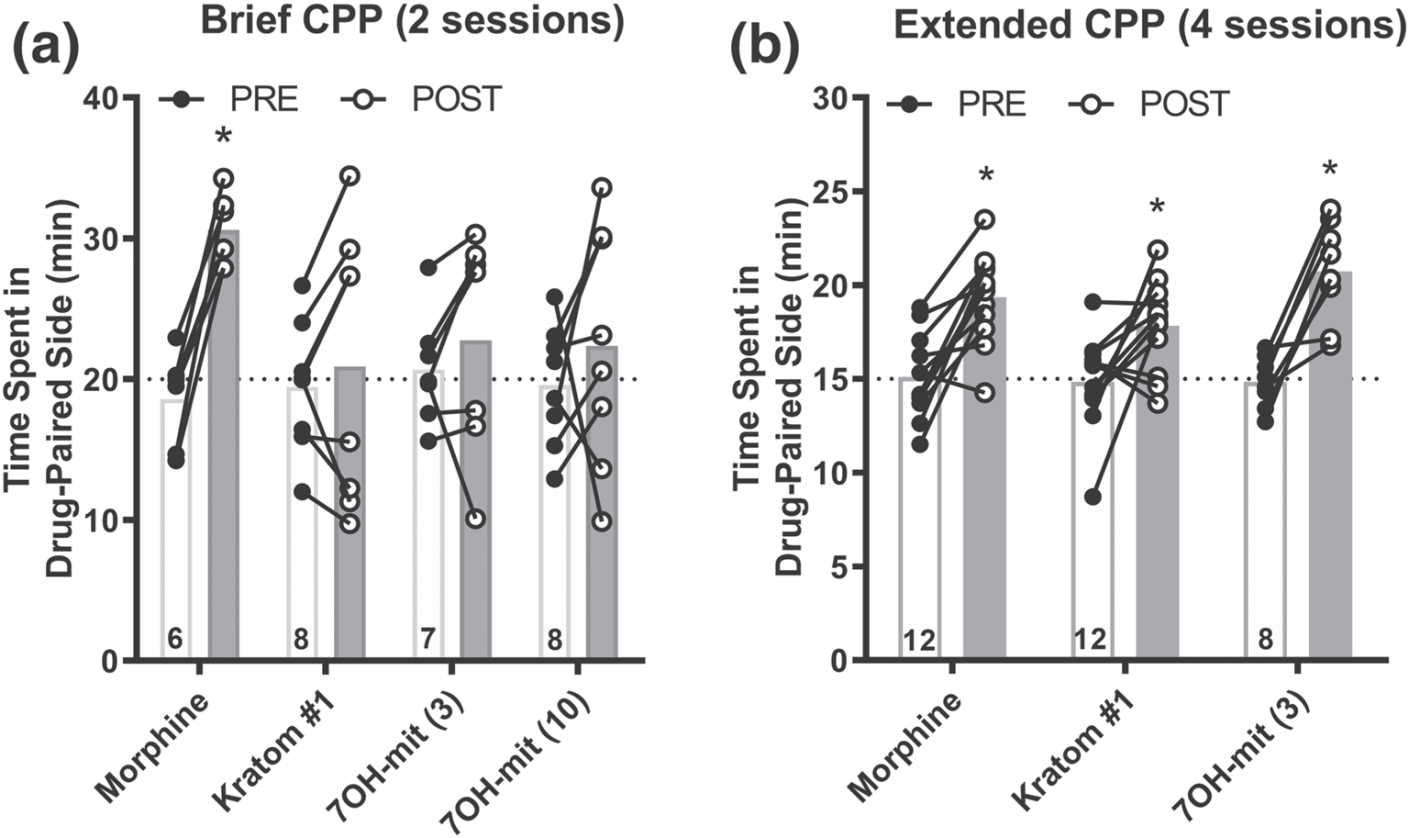
Mice will develop conditioned place preference for kratom and 7‐hydroxymitragynine but require more conditioning sessions than morphine. Male C56BL/6 wild‐type mice were conditioned to vehicle and 6 mg·kg^−1^ of morphine, 30 mg·kg^−1^ of kratom #1, and 3 or 10 mg·kg^−1^ of 7‐hydroxymitragynine (7‐OH‐mit) for 2 days (Brief CPP). Pre‐ and post‐conditioning exploration of the drug‐paired chamber was recorded over 40 min (a). Male C56BL/6 wild‐type mice were alternately conditioned for four times to vehicle and four times to 6 mg·kg^−1^ of morphine, 30 mg·kg^−1^ of kratom #1, or 3 mg·kg^−1^ of 7‐hydroxymitragynine over the course of 2 weeks (Extended CPP). Pre‐ and post‐conditioning exploration of the drug‐paired chamber was recorded over 30 min (b). See Section 3 for statistical analysis

Opioids that activate μOPs, even those that are G protein‐biased such as TRV130 (Altarifi et al., 2017; Austin Zamarripa et al., 2018), are rewarding (Fields & Margolis, 2015). We next assessed whether male mice will develop place preference to kratom or 7‐hydroxymitragynine using morphine as the positive control. In our brief CPP paradigm (two conditioning sessions), only 6 mg·kg^−1^ of morphine (*n* = 6) produced significant place preference (as determined by increased time spent on the drug‐paired side after conditioning; Figure 5a). Surprisingly, 30 mg·kg^−1^ of kratom extract #1 (*n* = 8), and 3 (*n* = 7) and 10 mg·kg^−1^ (n = 8) of 7‐hydroxymitragynine did not produce statistically significant increases in the amount of time spent on the drug‐paired side in the short CPP paradigm (Figure 5a). CPP magnitude can be enhanced for many substances of abuse by increasing the drug exposures (Lett, 1989). When extending the number of conditioning sessions from two to four, place preference was established for 30 mg·kg^−1^ of kratom extract #1 (*n* = 12), 3 mg·kg^−1^ of 7‐hydroxymitragynine (*n* = 8) and 6 mg·kg^−1^ of morphine (*n* = 12; Figure 5b).

### 3.9 Synthetic G protein‐biased opioids reduce alcohol intake with relatively limited rewarding effects

**Figure 6:**
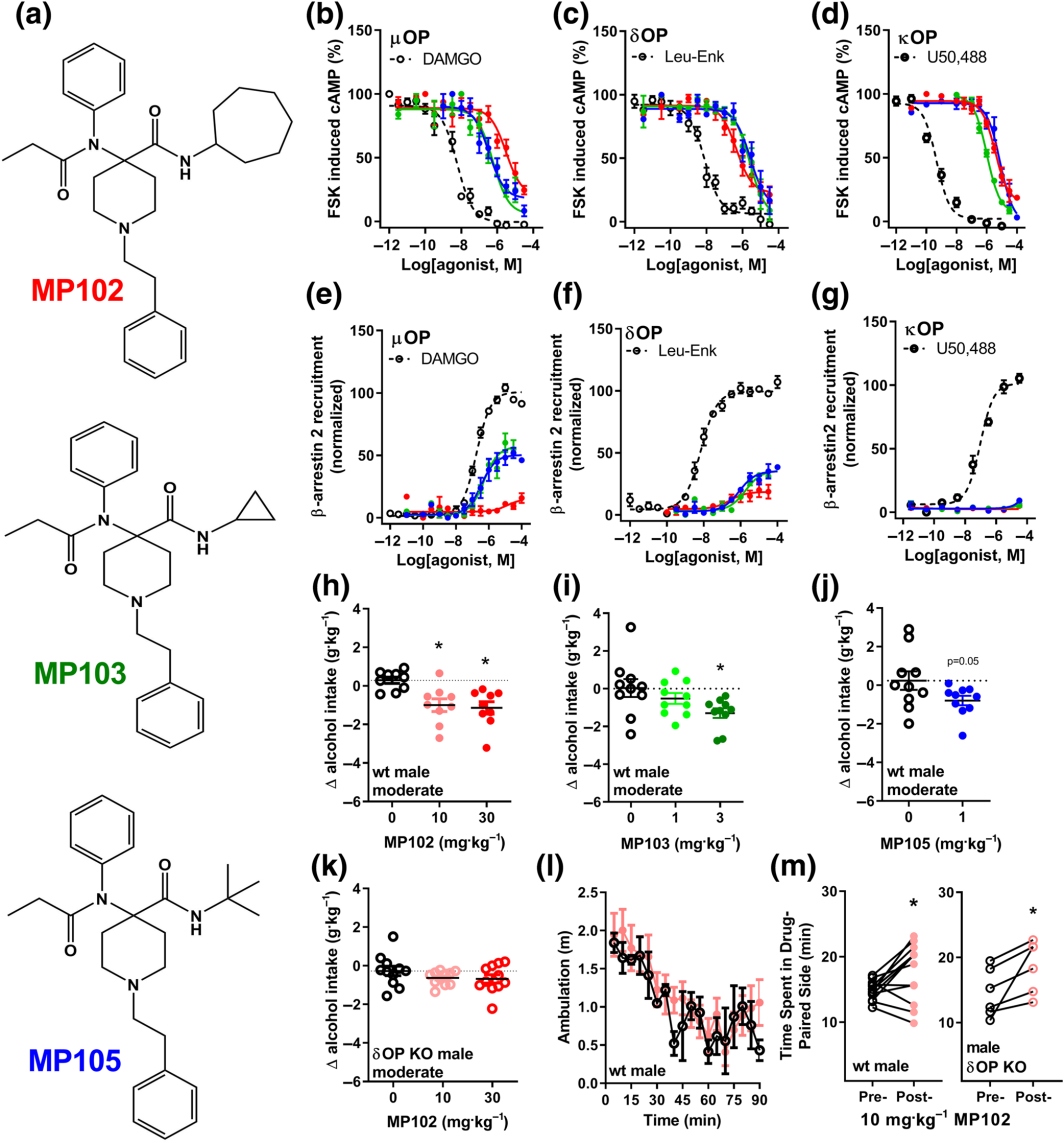
Synthetic G protein‐biased opioids reduce alcohol intake in mice. Chemical structures of MP102, MP103, and MP105 (a). MP102 (red ●), MP103 (green ▲), and MP105 (blue ■) reduced forskolin (FSK)‐induced cAMP production in HEK293 cells expressing μOP (b), δOP (c), or κOP (d), normalized to the relevant positive controls (dotted lines) DAMGO (μOP), leu‐enkephalin (δOP), and U50,488 (κOP). MP102 (red ●), only weakly recruited β‐arrestin 2 compared to MP103 (green ▲) and MP105 (blue ■) in CHO‐μOP (e), CHO‐δOP (f), or U2OS‐κOP (g) PathHunter cells normalized to the relevant positive controls (dotted lines). Systemic (i.p.) injection of MP102 (h) and MP103 (i) dose‐dependently decreased alcohol intake in male mice. Systemic injection of MP105 (1 mg·kg^−1^, i.p.) did not significantly decrease 10% alcohol intake (j). Systemic (i.p.) injection of MP102 did not reduce 10% alcohol intake in δOP knockout (KO) male mice (k). MP102 (10 mg·kg^−1^, i.p.) did not alter locomotor activity of male mice (l), but mice showed conditioned place preference to 10 mg·kg^−1^ of MP102 (m). See Section 3 for statistical analysis

Given that kratom and 7‐hydroxymitragynine still produced CPP, we next assessed whether an alternative drug that displayed selectivity for δOP over μOP would be more beneficial in reducing alcohol intake and produce fewer adverse side effects. We characterized a series of https://www.guidetopharmacology.org/GRAC/LigandDisplayForward?ligandId=10040‐amides (MP102, MP103, and MP105) previously shown to bind and activate both μOP and δOP (Varadi, Palmer, et al., 2015). We found that the three carfentanil‐amide opioids dose‐dependently inhibited cAMP production at μOP, δOP, and κOP (Figure 6a–c, Table 1). In agreement with the previously reported characterization (Varadi, Palmer, et al., 2015), MP102 was more potent at δOP than at μOP (Table 1). Similar to the kratom alkaloids, the carfentanil‐amide opioids did not strongly recruit β‐arrestin 2 at μOP, δOP, or κOP; MP102 displayed equally low β‐arrestin 2 recruitment efficacy at μOP and δOP; however, MP103 and MP105 were more efficacious at μOP than at δOP (Figure 6d–f, Table 1). In vivo, we found that MP102 and MP103 dose‐dependently reduced alcohol intake in male wild‐type mice (Figure 6g,h) with effective doses of 10 and 30 mg·kg^−1^ for MP102 and a dose of 3 mg·kg^−1^ for MP103. A dose of 1 mg·kg^−1^ of MP105 did not significantly decrease alcohol intake; however, at this dose, mice displayed abnormal head twitching behaviour; therefore, we refrained from testing any higher doses (Figure 6i). Similar to 7‐hydroxymitragynine, the ability of MP102 to reduce alcohol intake was dependent on the presence of δOP, as neither 10 nor 30 mg·kg^−1^ of MP102 was able to significantly reduce alcohol intake in δOP knockout mice (Figure 6j). The effective dose of 10 mg·kg^−1^ of MP102 (*n* = 8) did not alter the locomotor activity of male wild‐type mice (Figure 6k). In the extended CPP paradigm, we observed that mice injected with 10 mg·kg^−1^ of MP102 spent significantly more time in the drug‐paired side, an effect which persisted in δOP knockout mice.

## 4 DISCUSSION

Prior research regarding kratom and its alkaloids has emphasized the antinociceptive properties of 7‐hydroxymitragynine and kratom‐derived alkaloid synthetic derivatives, such as mitragynine and pseudoindoxyls (Matsumoto et al., 2006; Matsumoto et al., 2008; Takayama et al., 2002; Varadi et al., 2016) because these alkaloids appear to be G protein‐biased at the μOP, a characteristic potentially crucial for novel analgesic drug development. For example, recently developed G protein‐biased μOP agonists TRV130/oliceridine and https://www.guidetopharmacology.org/GRAC/LigandDisplayForward?ligandId=9286 supposedly increased the therapeutic window of μOP analgesics (DeWire et al., 2013; Manglik et al., 2016; Singla et al., 2017; Soergel et al., 2014; Viscusi et al., 2016), although recent reports have raised doubts on the robustness of these findings (Austin Zamarripa et al., 2018; Hill et al., 2018). In these studies, and in agreement with our findings, limited β‐arrestin 2 recruitment was observed at the μOP for both mitragynine and 7‐hydroxymitragynine compared with G protein activity (Kruegel et al., 2016; Varadi et al., 2016). By comparing G protein‐mediated cAMP inhibition with β‐arrestin 2 recruitment, our current results suggested that this functional selectivity of kratom alkaloids also holds true at the δOP and κOP. It is noteworthy that in our hands, 7‐hydroxymitragynine acted as a partial agonist at the δOP rather than as an antagonist as previously reported (Kruegel et al., 2016; Varadi et al., 2016). This discrepancy is possibly due to the use of different cellular assays to measure G protein activation: specifically, GTPγS and BRET assays versus inhibition of adenylyl cyclase mediated cAMP production (as used in this study). The cAMP assay may amplify signals such that a partial agonist in the GTPγS assay displays full agonism or a compound with no detectable activity in the GTPγS assay may display partial agonism in the cAMP assay. Supporting our *in vitro* finding of δOP agonism for 7‐hydroxymitragynine, 7‐hydroxymitragynine‐induced inhibition of cAMP production in cells expressing the δOP was attenuated using the δOR selective antagonist naltrindole (Figure S10).

The calculated bias factor of 0.44 for 7‐hydroxymitragynine at the dOP, is suggestive of bias towards β‐arrestin 2 seemingly in spite of a weak efficacy of β‐arrestin 2 recruitment. It appears that for 7‐hydroxymitragynine the β‐arrestin 2 bias was driven by a relatively strong potency for β‐arrestin 2 recruitment (Table 1). However, we have previously shown that β‐arrestin 2 recruitment efficacy (between 36% and 142%) strongly correlated with modulation of alcohol intake by δOP selective agonists (Chiang et al., 2016). This correlation holds true for both 7‐hydroxymtraginine and MP102 and extends the lower end of the correlation window to 14% β‐arrestin 2 recruitment efficacy (Figure S11).

A major concern with opioid agonists, particularly those with strong activity at μOP, is the development of physical and psychological dependence. There is a strong correlation between opioid self‐administration and opioid CPP (Morgan & Christie, 2011; Tzschentke, 2007). CPP to morphine is dependent on μOP expression (Matthes et al., 1996) but is enhanced in the absence of β‐arrestin 2 (Bohn et al., 2003), which would suggest that a G protein‐biased μOP agonists would have rewarding properties. Indeed, rats and mice develop CPP for G protein‐biased mitragynine and 7‐hydroxymitragynine (Matsumoto et al., 2008; Sufka et al., 2014). A recent rat self‐administration study found that 7‐hydromitragynine, but not mitragynine, could substitute for morphine. Interestingly, this response was blocked by a μOP antagonist as well as by the δOP antagonist naltrindole, although the naltrindole dose was relatively high and may not have been δOP specific (Hemby, McIntosh, Leon, Cutler, & McCurdy, 2018). Our results were in agreement with the published findings that mice display CPP for kratom and 7‐hydroxymitragyninine; however, we found that two conditioning sessions were insufficient to robustly produce kratom or 7‐hydroxymitragynine CPP in mice. In contrast, two conditioning sessions were sufficient to produce morphine CPP (Figure 5). In fact, even a single dose of morphine produces CPP (Bardo & Neisewander, 1986). However, increasing the number of conditioning sessions to four revealed CPP for 7‐hydroxymitragynine and kratom extract #1. Thus, as predicted based on the enhanced morphine CPP in β‐arrestin 2 knockout mice (Bohn et al., 2003), kratom and 7‐hydromytraginine were not strongly rewarding but did indeed possess rewarding properties.

In the “brief CPP” assay, we noticed that some mice developed conditioned place aversion to kratom extract #1 and 7‐hydroxymitragynine (see Figure 5a, points with negative slope). It is possible that this aversion was mediated by alkaloid activity at κOP. The aversive, dysphoric effects of κOP agonists have been associated with β‐arrestin 2 mediated p38 signalling by κOP (Bruchas et al., 2007; Land et al., 2009). However, a recent study revealed that κOP agonists still produced conditioned place aversion in β‐arrestin 2 knockout mice (White et al., 2015) and a number of studies have found that G protein‐biased κOP agonists still induced conditioned place aversion (Gupta et al., 2016; Spetea et al., 2017; White et al., 2015). Thus, it is possible that one mechanism by which kratom produced less pronounced CPP was via action at κOP‐associated dysphoria. κOPs and their endogenous opioid dynorphins have been associated with the negative affect of alcohol intake and withdrawal (Walker & Koob, 2008). In this study, we did not investigate if κOP‐induced aversion by kratom or kratom alkaloids contributed to the mechanism by which these alkaloids reduced alcohol intake. In our study, the potent and μOP selective, G protein‐biased agonist TRV130 did not alter alcohol intake in mice at effective analgesic doses (DeWire et al., 2013), suggesting that G protein‐biased μOP activity did not drive the decreased alcohol consumption observed for kratom and its alkaloids. Additionally, at the tested doses, TRV130 is known to be reinforcing (Altarifi et al., 2017), which would further suggest that mice were not consuming less alcohol when administered kratom because of μOP‐reward pathway activation.

Several studies have shown that activation of δOPs can modulate alcohol intake (Alongkronrusmee, Chiang, & van Rijn, 2016) and thus, a synthetic opioid that preferentially activates δOP could minimize the impact of μOP‐mediated reward and κOP‐mediated aversion on the behavioural effects in the alcohol assays. Indeed, our characterization revealed that MP102 had the desired *in vitro* pharmacological profile of a G protein‐biased, δOP‐preferring agonist. In accordance with our hypothesis, MP102 reduced alcohol intake in wild type (but not δOP knockout mice) and did not significantly alter locomotor activity. In comparison to μOP agonists and in agreement with prior findings, non‐peptidic δOP agonists were less rewarding (Do Carmo et al., 2009; Negus, Gatch, Mello, Zhang, & Rice, 1998; Stevenson, Folk, Rice, & Negus, 2005; van Rijn, Brissett, & Whistler, 2012a), and while MP102 still produced CPP, this conditioning was less robust than that observed for 7‐hydroxymitragynine and appeared not mediated by δOP, as it was still present in δOP knockout mice.

In conclusion, we found that kratom alkaloids do not recruit β‐arrestin 2 at the μOP, δOP and κOP and can significantly reduce both moderate and binge alcohol intake in male mice and female mice. This pharmacological profile and effect on alcohol intake in rodents may explain why some find kratom useful to self‐medicate for alcohol use disorder. Yet, as we observed that kratom extract and 7‐hydroxymitragynine exhibited reinforcing properties, our study also highlights the risks associated with kratom use. Our results indicate that δOPs contributed to the efficacy of the kratom alkaloids to reduce alcohol intake, whereas the lack of efficacy for the G protein‐biased μOP agonist TRV130 to decrease alcohol intake argued against a major role for the μOP in this behavioural response. The ability of MP102, a synthetic G protein‐biased opioid with a preference for δOP, to reduce alcohol intake without affecting general locomotion or inducing (δOP‐mediated) CPP provides support for future efforts to produce G protein‐selective, δOP‐selective opioids for the treatment of alcohol use disorder, some of which could be plant‐derived still as well (Cassell et al., 2019).

## AUTHOR CONTRIBUTIONS

A.M.G., M.T.R., R.J.C., R.M.vR. carriedout data acquisition, data analysis, data visualization, writing, editing and proofreading of the manuscript. R.U. and S.M. provided kratom extract, kratomalkaloids and synthetic opioids, edited and proofread the manuscript. M.J.K. and K.L.M. assisted in data acquisition and proofread the manuscript. G.W.P., S.M.and R.M.vR. provided supervision and acquired funding.

## CONFLICT OF INTEREST

Both G.W.P. and S.M. are co‐founders of Sparian BioSciences. All authors report no other biomedical financial interests or potential conflicts of interests.

## DECLARATION OF TRANSPARENCY AND SCIENTIFIC RIGOUR

This Declaration acknowledges that this paper adheres to the principles for transparent reporting and scientific rigour of preclinical research as stated in the *BJP* guidelines for https://bpspubs.onlinelibrary.wiley.com/doi/abs/10.1111/bph.14207, and https://bpspubs.onlinelibrary.wiley.com/doi/abs/10.1111/bph.14206, and as recommended by funding agencies, publishers and other organisations engaged with supporting research.

## Supporting information

Table S1. LogR (τ/Ka) and confidence intervals for tested alkaloids and opioids
Table S2: 2‐way ANOVA values for alcohol intake, water intake and alcohol preference
Supplemental Table 1. Mice injected with kratom extract decrease moderate and binge alcohol intake.
Supplemental Table 2. Kratom alkaloids decrease alcohol intake, but differentially affect general locomotion (performed in males unless specified otherwise).
Supplemental Table 3. Conditioned place preference to kratom alkaloids.
Supplemental Table 4. Synthetic G protein‐biased opioids reduce alcohol intake with relatively limited rewarding effects.
Figure S1 Description of the alkaloid extraction procedure from kratom. Procedure for kratom alkaloid extract #1 from Maeng Da kratom (A), Procedure for kratom alkaloid extract #2 from Red Indonesian kratom (B).
Figure S2 Effect of kratom extracts on alcohol intake, water intake and alcohol preference. Dose dependent effects of kratom extract #1 in male (A), kratom extract #1 in female (B), kratom extract #2 in male (C) C57BL/6 mice on alcohol intake, water intake and alcohol preference.
Figure S3 Effect of kratom alkaloids and TRV130 on alcohol intake, water intake and alcohol preference.
Figure S4 Effect of carfentanil‐amides on alcohol intake, water intake and alcohol preference.
Figure S5 Kratom extract #2 decreases voluntary alcohol intake in male mice. Systemic administration (i.p.) of kratom extract 2 dose‐dependently decreases alcohol intake in wild‐type C57BL/6 male mice.
Figure S6. Kratom extracts display G protein bias at μOP, δOP or κOP. Comparison of morphine with kratom extract #1 and #2 for the ability to reduce forskolin (FSK)‐induced cAMP production in HEK293 cells expressing μOP (A), δOP (B) or κOP (C), normalized to the relevant positive controls (dotted lines, open circles) DAMGO (μOP), leu‐enkephalin (δOP) and U50,488 (κOP). Comparison of morphine (red filled circles) with kratom extract #1 (green triangles) and extract #2 (orange squares) for the ability to recruit β‐arrestin 2 in CHO‐μOP (D), CHO‐δOP (E) or U2OS‐κOP (F) PathHunter cells normalized to the relevant positive controls (dotted lines).
Figure S7. Kratom alkaloids display G protein bias at μOR, δOR or κOP
Figure S8. TRV130 displays limited potency at δOR or κOP.
**Figure S9. Dose dependent antinociception produced by systemic TRV130**. TRV130 injected at 1, 3 an d 10 mg/kg (s.c.) produced antinociception in male WT C57BL/6 mice 30 minutes post injection as measured on the tail‐flick assay. The experiment was carried out over 3 days in the same animals (*n*=10). Only 8 mice were used for the 10 mg/kg dose. Statistics were performed using a two‐way RM ANOVA: Treatment x dose F2,25 = 53, *p*<0.0001; Treatment F2,25 = 69, <0.0001; Dose F1,25 = 277, *p*<0.0001.
**Figure S10. The** **OP antagonist naltrindole blocks 7‐hydroxymitragynine‐induced inhibition of cAMP production.**
**Figure S11. Correlation between β‐arrestin 2 recruitment efficacy at** **OP and modulation of alcohol intake.**
