# Supplementary material for: G protein‐biased kratom‐alkaloids and synthetic carfentanil‐amide opioids as potential treatments for alcohol use disorder
Source: Br J Pharmacol. 2020 Jan 24;177(7):1497–513. doi: 10.1111/bph.14913 (PMC7060366; doi:10.1111/bph.14913)
Supplement: Supplementary file 1 — Table S1. LogR (τ/Ka) and confidence intervals for tested alkaloids and opioids Table S2: 2‐way ANOVA values for alcohol intake, water intake and alcohol preference Supplemental Table 1. Mice injected with kratom extract decrease moderate and binge alcohol intake. Supplemental Table 2. Kratom alkaloids decrease alcohol intake, but differentially affect general locomotion (performed in males unless specified otherwise). Supplemental Table 3. Conditioned place preference to kratom alkaloids. Supplemental Table 4. Synthetic G protein‐biased opioids reduce alcohol intake with relatively limited rewarding effects. Figure S1 Description of the alkaloid extraction procedure from kratom. Procedure for kratom alkaloid extract #1 from Maeng Da kratom (A), Procedure for kratom alkaloid extract #2 from Red Indonesian kratom (B). Figure S2 Effect of kratom extracts on alcohol intake, water intake and alcohol preference. Dose dependent effects of kratom extract #1 in male (A), kratom extract #1 in female (B), kratom extract #2 in male (C) C57BL/6 mice on alcohol intake, water intake and alcohol preference. Figure S3 Effect of kratom alkaloids and TRV130 on alcohol intake, water intake and alcohol preference. Figure S4 Effect of carfentanil‐amides on alcohol intake, water intake and alcohol preference. Figure S5 Kratom extract #2 decreases voluntary alcohol intake in male mice. Systemic administration (i.p.) of kratom extract 2 dose‐dependently decreases alcohol intake in wild‐type C57BL/6 male mice. Figure S6. Kratom extracts display G protein bias at μOP, δOP or κOP. Comparison of morphine with kratom extract #1 and #2 for the ability to reduce forskolin (FSK)‐induced cAMP production in HEK293 cells expressing μOP (A), δOP (B) or κOP (C), normalized to the relevant positive controls (dotted lines, open circles) DAMGO (μOP), leu‐enkephalin (δOP) and U50,488 (κOP). Comparison of morphine (red filled circles) with kratom extract #1 (green triangles) and extract #2 (orange squares) [file BPH-177-1497-s001.pdf]

Supplementary Materials

Table S1: LogR (tau/Ka) and confidence intervals for tested alkaloids and opioids

Compounds	cAMP		β-arrestin 2	
μOP	LogR	95%CI	LogR	95%CI
DAMGO	8.185	7.939 to 8.427	6.764	6.684 to 6.841
Kratom #1	5.378	5.159 to 5.593	ND	ND
Kratom #2	5.594	5.337 to 5.847	ND	ND
Morphine	8.538	8.175 to 8.889	6.095	Ambiguous
Mitragynine	5.536	5.302 to 5.769	ND	ND
7-OH-mitragynine	5.123	4.889 to 5.343	ND	ND
Speciogynine	5.423	5.210 to 5.629	ND	ND
Paynantheine	7.355	6.995 to 7.705	ND	ND
TRV130	7.559	7.275 to 7.831	8.213	Ambiguous
MP102	5.26	5.011 to 5.503	4.601	Ambiguous
MP103	6.102	5.840 to 6.363	6.144	5.922 to 6.367
MP105	5.999	5.741 to 6.253	6.269	6.006 to 6.538
δOP	LogR	95%CI	LogR	95%CI
Leu-enkephalin	8.028	7.807 to 8.241	8.164	8.079 to 8.247
Kratom #1	4.36	4.075 to 4.608	ND	ND
Kratom #2	4.911	4.622 to 5.179	ND	ND
Morphine	5.719	5.465 to 5.970	ND	ND
Mitragynine	4.393	4.099 to 4.646	ND	ND
7-OH-mitragynine	4.841	4.600 to 5.065	ND	ND
Speciogynine	4.756	4.464 to 5.025	ND	ND
Paynantheine	5.535	5.337 to 5.730	ND	ND
TRV130	3.954	3.478 to 4.306	ND	ND
MP102	5.989	5.728 to 6.245	5.838	4.868 to 6.829
MP103	5.667	5.430 to 5.903	5.227	4.748 to 5.716
MP105	5.536	5.324 to 5.745	5.532	5.026 to 6.042
κOP	LogR	95%CI	LogR	95%CI
U50,488	8.649	8.445 to 8.846	6.947	ND
Kratom #1	4.375	4.056 to 4.658	ND	ND
Kratom #2	6.503	6.195 to 6.806	ND	ND
Morphine	6.468	6.144 to 6.785	ND	ND
Mitragynine	4.32	3.997 to 4.602	ND	ND
7-OH-mitragynine	5.368	3.577 to 4.355	ND	ND
Speciogynine	4.013	4.750 to 5.291	ND	ND
Paynantheine	5.028	5.147 to 5.587	ND	ND
TRV130	4.098	3.647 to 4.454	ND	ND
MP102	5.24	5.098 to 5.379	ND	ND
MP103	5.848	5.690 to 6.004	ND	ND
MP105	5.148	5.013 to 5.281	ND	ND

Table S2: 2-way ANOVA values for alcohol intake, water intake and alcohol preference

		Dose	*p*	Time	*p*	Dose x Time	*p*
Kratom extract #1 male	alcohol	F(2,24) = 32.05	<0.05	F(1,24) = 15.55	<0.05	F(2,24) = 32.05	<0.05
	water	F(2,24) = 4.463	<0.05	F(1,23) = 1.681		F(2,23) = 0.6609	
	preference	F(2,24) = 12.45	<0.05	F(1,23) = 5.083	<0.05	F(2,23) = 6.063	<0.05
Kratom extract #2 male	alcohol	F(2,33) = 0.3255		F(1,33) = 35.52	<0.05	F(2,33)=7.185	<0.05
	water	F(2,33) = 2.760		F(1,33) = 5.462	<0.05	F(2,33)=0.211	
	preference	F(2,33) = 1.967	0.1559	F(1,33) = 0.8999		F(2,33)=0.001457	
Kratom extract #1 female	alcohol	F(2,27) = 6.818	<0.05	F(1,27)=40.41	<0.05	F(2,27)=15.31	<0.05
	water	F(2,27) = 0.9793		F(1,27)=1.558		F(2,27)=8.165	<0.05
	preference	F(2,27) = 3.525	<0.05	F(1,27)=6.983	<0.05	F(2,27)=5.685	<0.05
mitragynine male	alcohol	F(3,32)=6.646	<0.05	F(1,32)=12.64	<0.05	F(3,32)=4.186	<0.05
	water	F(3,32)=2.917	<0.05	F(1,32)=6.356	<0.05	F(3,32)=5.493	<0.05
	preference	F(3,32)=7.338	<0.05	F(1,32)=10.45	<0.05	F(3,32)=7.9	<0.05
Paynantheine male	alcohol	F(2,27) = 9.858	<0.05	F(1,27)=0.5897		F(2,27)=12.81	<0.05
	water	F(2,27) = 1.886		F(1,27)=0.2371		F(2,27)=1.365	
	preference	F(2,27) = 0.1286		F(1,27)=5.606	<0.05	F(2,27)=5.327	<0.05
speciogynine male	alcohol	F(2,27) = 5.142	<0.05	F(1,27)=18.70	<0.05	F(2,27)=36.52	<0.05
	water	F(2,27) = 3.601		F(1,27)=9.087	<0.05	F(2,27)=0.6933	
	preference	F(2,27) = 1.087		F(1,27)=0.4053		F(2,27)=5.649	<0.05
7-OH-mitragyinine male	alcohol	F(3,32)=0.832		F(1,32)=12.73	<0.05	F(3,32)=9.589	<0.05
	water	F(3,32)=0.09804		F(1,32)=0.2047		F(3,32)=8.913	<0.05
	preference	F(3,32)=1.250		F(1,32)=2.487		F(3,32)=0.5309	
7-OH-mitragyinine male δOP KO	alcohol	F(2,30)=0.2055		F(1,30)=9.445	<0.05	F(2,30)=1.974	
	water	F(2,30)=4.845	<0.05	F(1,30)=6.905	<0.05	F(2,30)=0.5391	
	preference	F(2,30)=3.740	<0.05	F(1,30)=1.241		F(2,30)=1.642	
7-OH-mitragyinine female	alcohol	F(1,22) = 0.6339		F(1,22) = 29.02	<0.05	F(1,22) = 16.13	<0.05
	water	F(1,22) = 1.747		F(1,22) = 1.236		F(1,22) = 1.269	
	preference	F(1,22) = 0.1126		F(1,22) = 0.0057		F(1,22) = 2.361	
TRV130 male	alcohol	F(2,24)=0.047		F(1,24)=1.027		F(2,24)=1.656	
	water	F(2,24)=1.091		F(1,24)=0.7158		F(2,24)=2.436	
	preference	F(2,24)=0.3609		F(1,24)=0.1085		F(2,24)=0.9047	
MP102 male	alcohol	F(2,24)=1.180		F(1,24)=14.3	<0.05	F(2,24)=7.852	<0.05
	water	F(2,24)=1.003		F(1,24)=0.1165		F(2,24)=1.181	
	preference	F(2,24)=0.248		F(1,24)=2.561		F(2,24)=1.015	
MP103 male	alcohol	F(2,27) = 3.601		F(1,27)=9.087	<0.05	F(2,27)=0.6933	
	water	F(2,27) = 4.514	<0.05	F(1,27)=1.979		F(2,27)=10.19	<0.05
	preference	F(2,27) =0.5811		F(1,27)=1.244		F(2,27)=7.087	<0.05
MP105 male	alcohol	F(1,18) = 3.871		F(1,18) = 0.8087		F(1,18) = 3.612	
	water	F(1,18) = 1.646		F(1,18) = 4.432	<0.05	F(1,18) = 4.159	<0.05
	preference	F(1,18) = 0.0027		F(1,18) = 1.367		F(1,18) = 0.3563	
MP102 male δOP KO	alcohol	F(2,30)= 1.076		F(1,30)=20.77	<0.05	F(2,30)=1.251	
	water	F(2,30)=9.843	<0.05	F(1,30)=3.705	<0.05	F(2,30) = 8.650	<0.05
	preference	F(2,30)=8.721	<0.05	F(1,30)=0.1557		F(2,30) = 10.06	<0.05

Supplemental Table 1. Mice injected with kratom extract decrease moderate and binge alcohol intake.			
	**Test**	**df**	**Significance**
**Moderate alcohol intake**			
Male, kratom extract #1	One-way ANOVA	2, 24	F = 15.0<br>p <0.05
Female, kratom extract #1	One-way ANOVA	2, 33	F = 2.12
Male, kratom extract #2	One-way ANOVA	2, 27	F = 11.59<br>p <0.05
**Binge alcohol intake**			
Male, kratom extract #1	Unpaired t test	8	p <0.05
Female, kratom extract #1	Unpaired Mann-Whitney U-test	8	p <0.05
**Locomotor activity**			
Male, kratom extract #1	Unpaired Mann-Whitney U-test	26	p <0.05
Female, kratom extract #1	Unpaired t test with Welsh's correction	8	p <0.05
**Locomotor activity by time**			
Male, kratom extract #1	Two-way ANOVA	17, 408	F = 6.226<br>p <0.05
Female, kratom extract #1	Two-way ANOVA	17, 238	F = 2.25<br>p <0.05
**Sex differences**			
Moderate alcohol intake, baseline	Unpaired t test	19	P < 0.05
Moderate alcohol intake, Kratom extract #1	Two-way ANOVA, interaction	2, 57	F = 1.75
Binge alcohol intake, baseline	Unpaired t test	14	
Binge alcohol intake, kratom extract #1	Two-way ANOVA, interaction	1, 28	F = 0.46
Locomotor effects of kratom extract #1	Two-way ANOVA, interaction	1, 38	F = 9.10<br>p <0.05

Supplemental Table 2. Kratom alkaloids decrease alcohol intake, but differentially affect general locomotion (performed in males unless specified otherwise).			
	**Test**	**df**	**Significance**
**Alcohol intake, wild-type**			
Mitragynine	One-way ANOVA	3, 32	F = 5.15<br>p <0.05
Paynantheine	One-way ANOVA	2, 27	F = 12.5<br>p <0.05
Speciogynine	One-way ANOVA	2, 24	F = 32.9<br>p <0.05
7-hydroxymitragynine	One-way ANOVA	3, 32	F = 10.4<br>p <0.05
Females, 7-hydroxymitragynine	Paired t test		p <0.05
TRV130	One-way ANOVA	2, 24	F = 1.511
**Locomotor activity, wild-type**			
Kratom alkaloids	One-way ANOVA	4, 37	F = 16.48<br>p <0.05
Female, 7-hydroxymitragynine	Unpaired t test with Welch's correction	8	p <0.05
**Alcohol intake, δOP knockout**			
7-hydroxymitragynine	One-way ANOVA	2, 30	F = 1.77
**Locomotor activity, δOP knockout**			
7-hydroxymitragynine	Unpaired Mann-Whitney U-test	18	p <0.05
**Peak locomotor activity, δOP knockout**			
7-hydroxymitragynine	Two-way ANOVA, time	17, 255	F = 46.99<br>p <0.05
**Sex differences, δOP knockout**			
Locomotor activity, 7-hydroxymitragynine	Two-way ANOVA, Interaction	1, 30	F = 0.96
Moderate alcohol intake, 7-hydroxymitragynine	Two-way ANOVA, interaction	1, 38	F = 4.96<br>p <0.05

Supplemental Table 3. Conditioned place preference to kratom alkaloids.

	Effect	df	Significance
**Brief CPP**			
Morphine	Drug	3, 25	F = 0.88
	Conditioning	1, 25	F = 12.36<br>$p < 0.05$
	Drug x conditioning	3, 25	F = 3.25<br>$p < 0.05$
	Matching	25, 25	F = 2.29<br>$p < 0.05$
**Extended CPP**			
Kratom alkaloids	Drug	2, 29	F = 1.97
	Conditioning	1, 29	F = 55.83<br>$p < 0.05$
	Drug x conditioning	2, 29	F = 1.89
	Matching	29, 29	F = 1.04

**Supplemental Table 4. Synthetic G protein-biased opioids reduce alcohol intake with relatively limited rewarding effects.**			
	**Test**	**df**	**Significance**
**Moderate alcohol intake, wild-type**			
MP102	One-way ANOVA	2, 24	F = 11.4<br>*p* <0.05
MP103	One-way ANOVA	2, 24	F = 4.2<br>*p* <0.05
MP105	Paired t test, Wilcoxon ranked	10	*p* <0.05
**Alcohol intake, δOP knockout**			
MP102	One-way ANOVA	2, 30	F = 1.2
**Locomotor activity, δOP knockout**			
**MP102**	Two-way ANOVA, effect of drug	1, 10	F = 0.25
**Extended CPP**			
Wild-type, MP102	Paired t test	11	*p* <0.05
δOP knockout, MP102	Paired t test	5	*p* <0.05

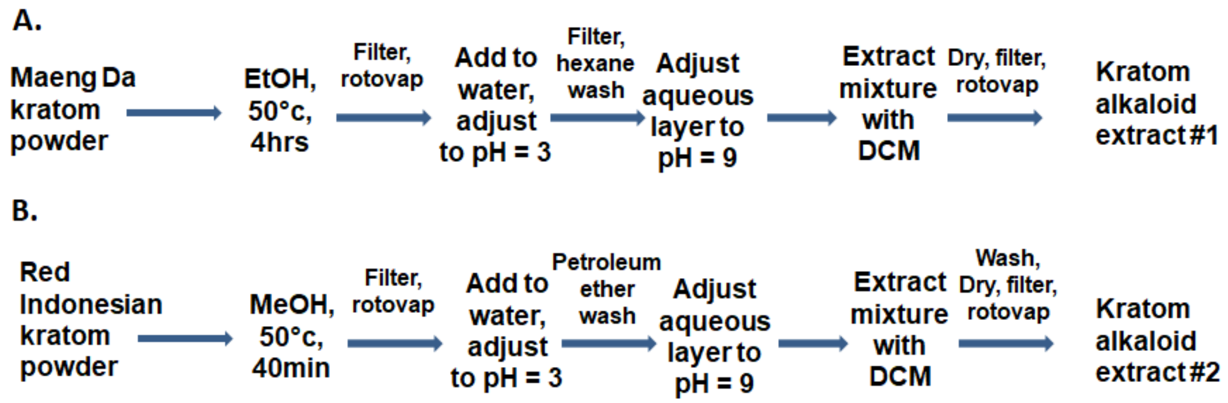

Figure S1 Description of the alkaloid extraction procedure from kratom. Procedure for kratom alkaloid **extract #1** from Maeng Da kratom (**A**), Procedure for kratom alkaloid extract #2 from Red Indonesian kratom (**B**).

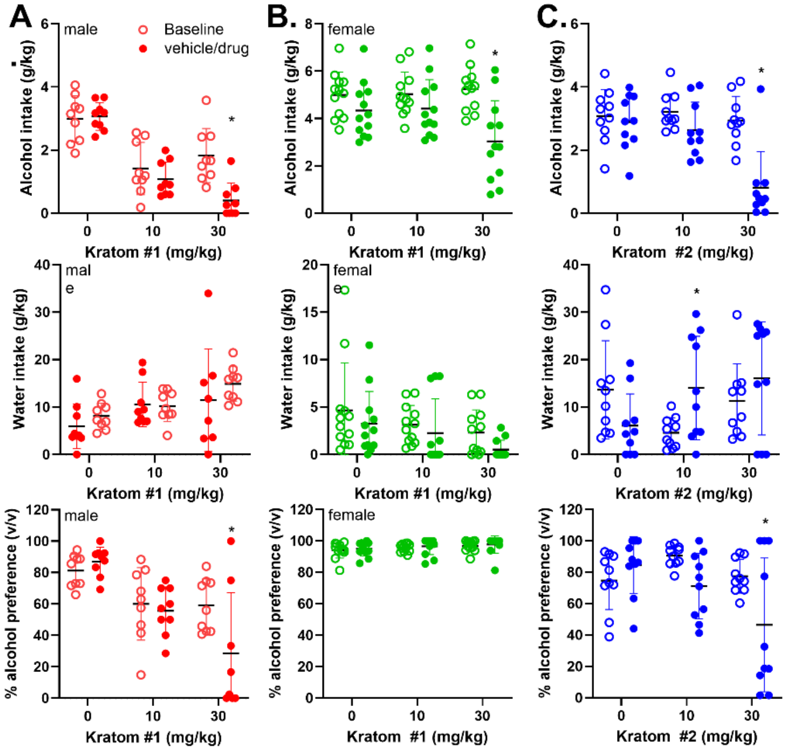

Figure S2 Effect of kratom extracts on alcohol intake, water intake and alcohol preference. Dose dependent effects of kratom extract #1 in male (A), kratom extract #1 in female (B), kratom extract #2 in male (C) C57BL/6 mice on alcohol intake, water intake and alcohol preference.

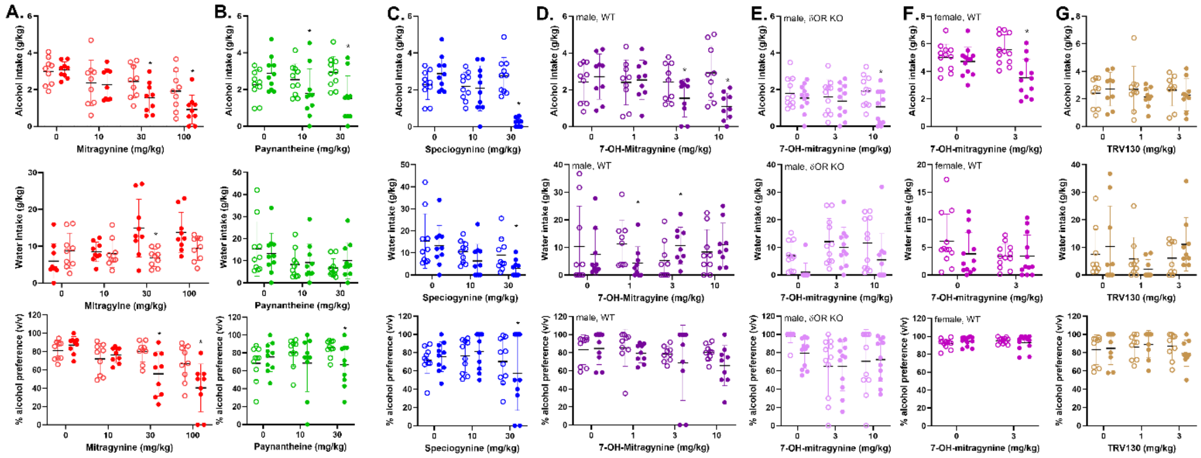

Figure S3 Effect of kratom alkaloids and TRV130 on alcohol intake, water intake and alcohol preference. Dose dependent effects of mitragynine in male (A), paynantheine in male (B), speciogynine in male (C), 7-hydroxymitragynine in male (D), 7-hydroxymitragynine in male δOP KO (E) 7-hydroxymitragynine in female (F) TRV130 in male (G) C57BL/6 mice on alcohol intake, water intake and alcohol preference.

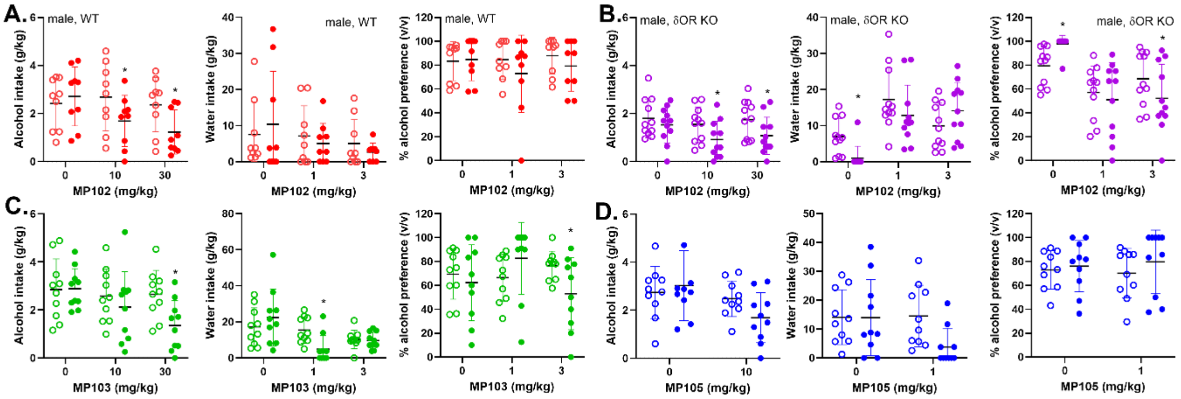

Figure S4 Effect of carfentanil-amides on alcohol intake, water intake and alcohol preference. Dose dependent effects of MP102 in male (A), MP102 in male δOP KO (B), MP103 in male (C) and MP105 in male (D) C57BL/6 mice on alcohol intake, water intake and alcohol preference.

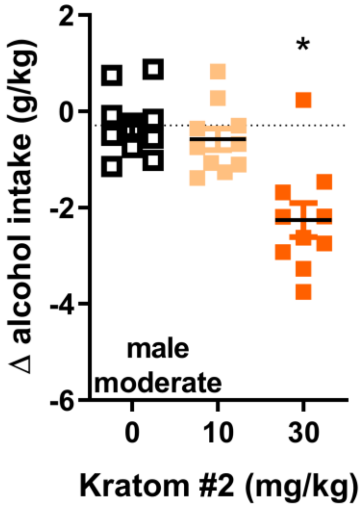

Figure S5 Kratom extract #2 decreases voluntary alcohol intake in male mice. Systemic administration (i.p.) of kratom extract 2 dose-dependently decreases alcohol intake in wild-type C57BL/6 male mice.

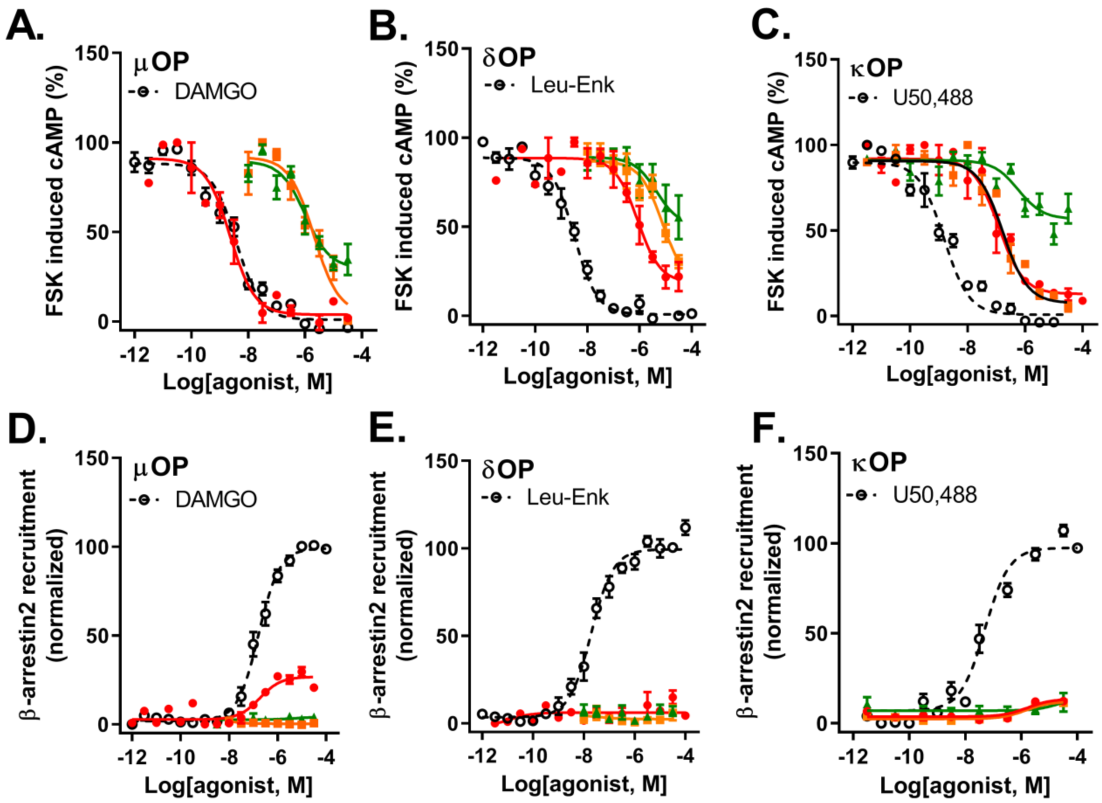

Figure S6. Kratom extracts display G protein bias at μOP, δOP or κOP. Comparison of morphine with kratom extract #1 and #2 for the ability to reduce forskolin (FSK)-induced cAMP production in HEK293 cells expressing μOP (**A**), δOP (**B**) or κOP (**C**), normalized to the relevant positive controls (dotted lines, open circles) DAMGO (μOP), leu-enkephalin (δOP) and U50,488 (κOP). Comparison of morphine (red filled circles) with kratom extract #1 (green triangles) and extract #2 (orange squares) for the ability to recruit β-arrestin 2 in CHO-μOP (**D**), CHO-δOP (**E**) or U2OS-κOP (**F**) PathHunter cells normalized to the relevant positive controls (dotted lines).

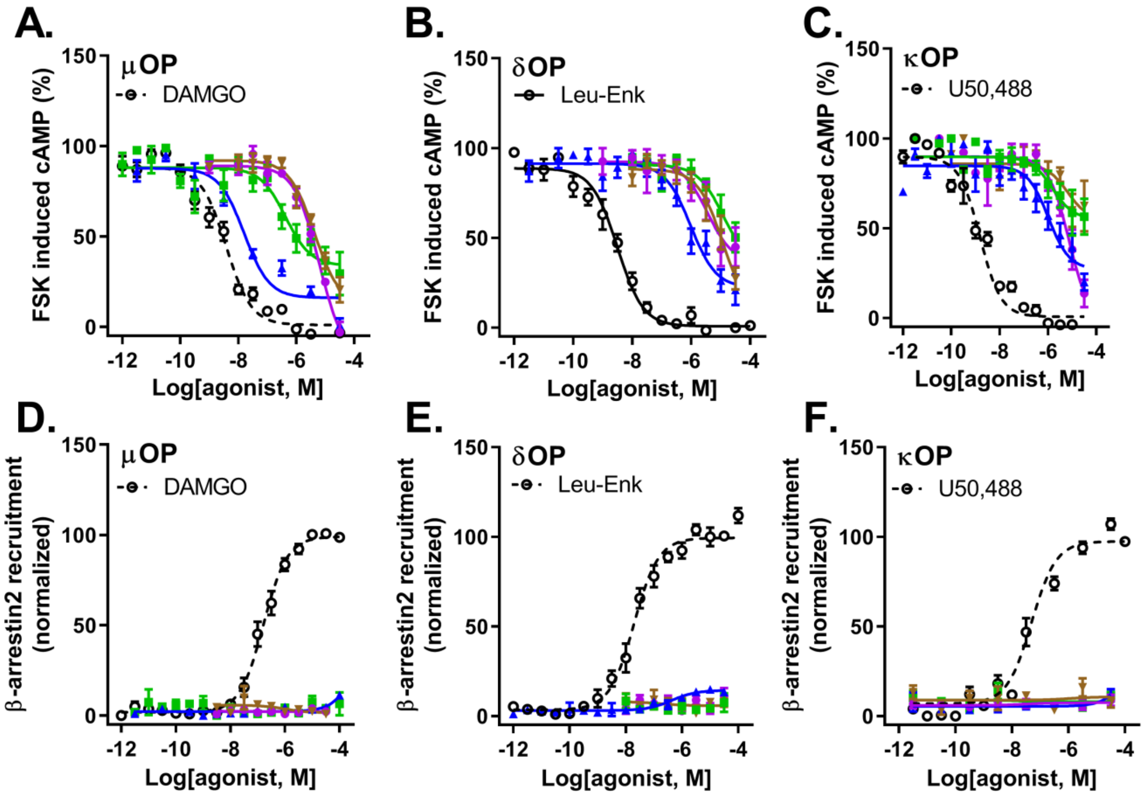

Figure S7. Kratom alkaloids display G protein bias at μOR, δOR or κOP Mitragynine (green ■), 7-OH-mitragynine (blue ▲), paynantheine (purple ●) and speciogynine (brown▼) reduce forskolin (FSK)-induced cAMP production in HEK293 cells expressing μOR (**A**), δOR (**B**) or κOR (**C**), normalized to the relevant positive controls (dotted lines) DAMGO (μOR), leu-enkephalin (δOR) and U50,488 (κOR). Mitragynine (green ■), 7-OH-mitragynine (blue ▲), paynantheine (purple ●) and speciogynine (brown▼) do not recruit β-arrestin 2 in CHO-μOP (**D**), CHO-δOR (**E**) or U2OS-κOR (**F**) PathHunter cells normalized to the relevant positive controls (dotted lines, open circles).

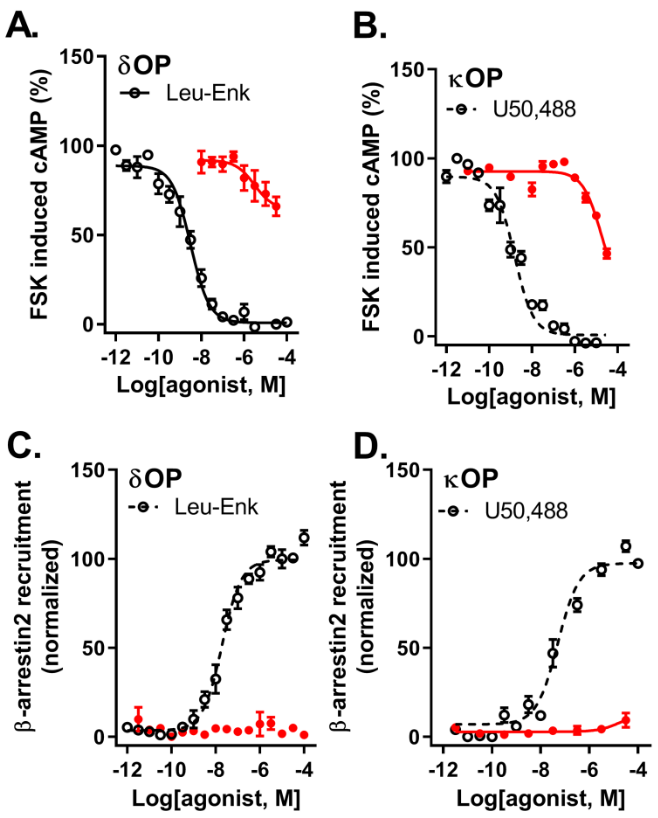

Figure S8. TRV130 displays limited potency at δOR or κOP. The G protein-biased µOR agonists TRV130 (red ●) reduces forskolin (FSK)-induced cAMP production in HEK293 cells expressing t δOP (**A**) or κOP (**B**) with low potency, normalized to the relevant positive controls (dotted lines), leu-enkephalin (δOP) and U50,488 (κOP). TRV130 (red ●) does not recruit β-arrestin 2 in, CHO-δOP (**C**) or U2OS-κOP (**D**) PathHunter cells normalized to the relevant positive controls (dotted lines).

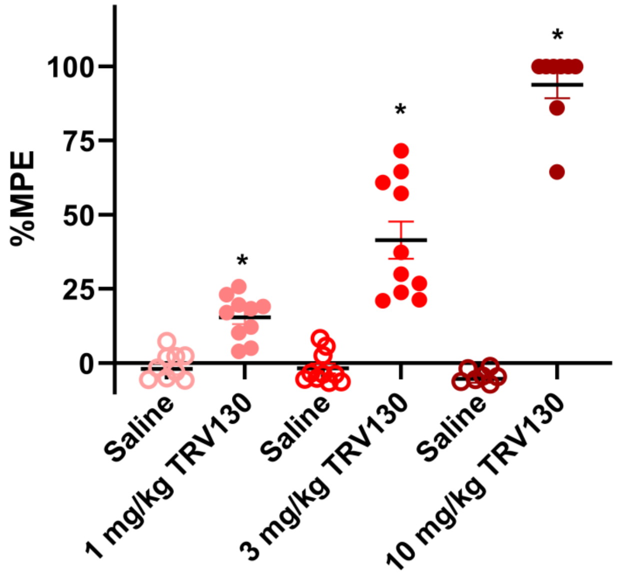

Figure S9. Dose dependent antinociception produced by systemic TRV130. TRV130 injected at 1, 3 an d 10 mg/kg (s.c.) produced antinociception in male WT C57BL/6 mice 30 minutes post injection as measured on the tail-flick assay. The experiment was carried out over 3 days in the same animals (n=10). Only 8 mice were used for the 10 mg/kg dose. Statistics were performed using a two-way RM ANOVA: Treatment x dose $F_{2,25} = 53$, $p<0.0001$; Treatment $F_{2,25} = 69$, $p<0.0001$; Dose $F_{1,25} = 277$, $p<0.0001$.

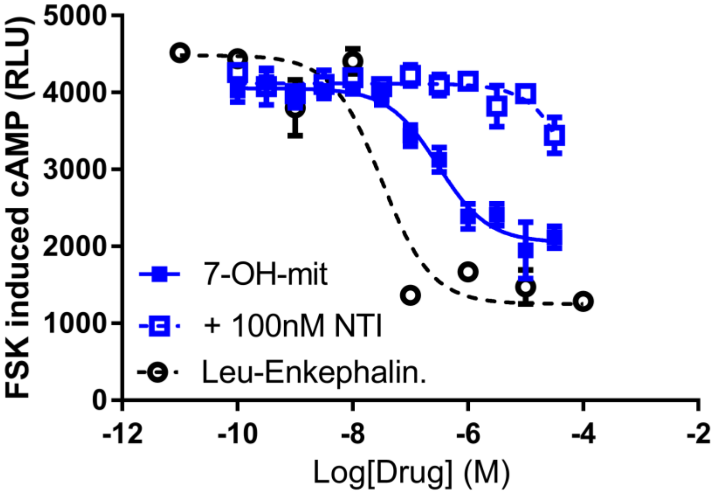

Figure S10. The δOP antagonist naltrindole blocks 7-hydroxymitragynine-induced inhibition of cAMP production. HEK293 cells, transiently expressing δOP and Glosensor were pre-incubated with 100 nM naltrindole for 10 minutes before stimulation with increasing concentration of 7-hydroxymitragynine. Experiments were ran in triplicate, and performed on three separate occasions. A representative curve is shown.

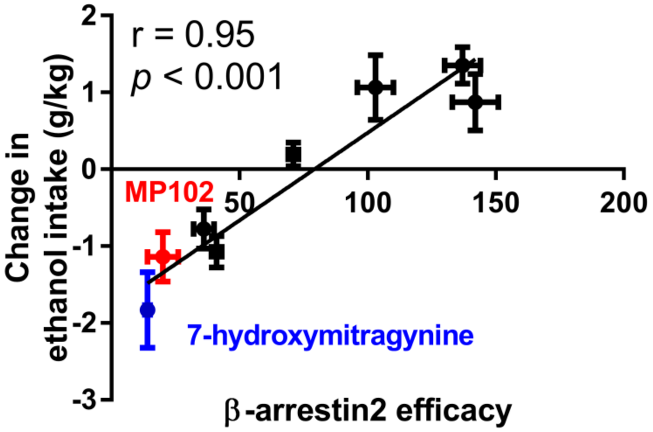

Figure S11. Correlation between β-arrestin 2 recruitment efficacy at δOP and modulation of alcohol intake. Addition of the very weak β-arrestin 2 recruiters MP102 and 7-hydroxymitragynine to the correlation previously established using the δOP selective agonists TAN-67, NIH11082, KNT127, ARM390, SNC80 and SNC162 (see *1*) further strengthens the hypothesis that β-arrestin 2 recruitment efficacy, not necessarily potency, impacts the directional response of a δOP agonist on alcohol intake.

1. T. Chiang, K. Sansuk, R. M. van Rijn, Beta-arrestin 2 dependence of delta opioid receptor agonists is correlated with alcohol intake. *Br J Pharmacol* **173**, 323-343 (2016).

.
